# Malate transported from chloroplast to mitochondrion triggers production of ROS and PCD in *Arabidopsis thaliana*

**DOI:** 10.1038/s41422-018-0024-8

**Published:** 2018-03-14

**Authors:** Yannan Zhao, Lilan Luo, Jiesi Xu, Peiyong Xin, Hongyan Guo, Jian Wu, Lin Bai, Guodong Wang, Jinfang Chu, Jianru Zuo, Hong Yu, Xun Huang, Jiayang Li

**Affiliations:** 1 State Key Laboratory of Plant Genomics and National Center for Plant Gene Research, Institute of Genetics and Developmental Biology, Chinese Academy of Sciences, Beijing, 100101 China; 2 University of Chinese Academy of Sciences, Beijing, 100049 China; 3 State Key Laboratory of Molecular Developmental Biology, Institute of Genetics and Developmental Biology, Chinese Academy of Sciences, Beijing, 100101 China; 4 Present Address: Department of Plant Science, Weizmann Institute of Science, Rehovot, 7610001 Israel

## Abstract

Programmed cell death (PCD) is a fundamental biological process. Deficiency in MOSAIC DEATH 1 (MOD1), a plastid-localized enoyl-ACP reductase, leads to the accumulation of reactive oxygen species (ROS) and PCD, which can be suppressed by mitochondrial complex I mutations, indicating a signal from chloroplasts to mitochondria. However, this signal remains to be elucidated. In this study, through cloning and analyzing a series of *mod1* suppressors, we reveal a comprehensive organelle communication pathway that regulates the generation of mitochondrial ROS and triggers PCD. We show that mutations in *PLASTIDIAL NAD-DEPENDENT MALATE DEHYDROGENASE* (*plNAD-MDH*), chloroplastic *DICARBOXYLATE TRANSPORTER 1* (*DiT1*) and *MITOCHONDRIAL MALATE DEHYDROGENASE 1* (*mMDH1*) can each rescue the ROS accumulation and PCD phenotypes in *mod1*, demonstrating a direct communication from chloroplasts to mitochondria via the malate shuttle. Further studies demonstrate that these elements play critical roles in the redox homeostasis and plant growth under different photoperiod conditions. Moreover, we reveal that the ROS level and PCD are significantly increased in malate-treated HeLa cells, which can be dramatically attenuated by knockdown of the human gene *MDH2*, an ortholog of *Arabidopsis mMDH1*. These results uncover a conserved malate-induced PCD pathway in plant and animal systems, revolutionizing our understanding of the communication between organelles.

## Introduction

Programmed cell death (PCD), a genetically controlled process of cell suicide, is a fundamental event of life, and originated before the advent of eukaryotes.^1^ Although similar attributes and common repressors have been found between plant and animal PCD,^2,3^ the uniqueness of plant PCD signaling pathways has emerged recently.^4,5^ PCD is now realized as essential for plant organogenesis and biotic/abiotic stress responses through its diverse functions in developmental patterning, cell differentiation, cell number regulation, and the hypersensitive response (HR).^6–10^ However, the understanding of different PCD signaling pathways in plants is still very limited.

Reactive oxygen species (ROS) provide a common thread and play multiple roles in many plant PCD systems.^4,11^ ROS can be generated from various subcellular sources, including mitochondria and chloroplasts. Chloroplasts are considered as the main source of ROS in the presence of light,^12^ and many types of plant PCD require functional chloroplasts and light.^4^ However, newly identified PCD components in mitochondria reveal the function of mitochondria in triggering and executing PCD in plants.^4^ ROS can act as PCD-inducing signaling molecules that trigger mitochondrial membrane permeabilization or the formation of the mitochondrial permeability transition pore (MPTP).^4,13^ ROS could also be elevated to a lethal level by cytochrome c release and the disruption of mitochondrial electron transport chain (mETC) in PCD execution.^4,14,15^ These findings demonstrate that both chloroplasts and mitochondria play essential roles in triggering and executing PCD. Communications between nucleus and organelles have been well-studied and these include anterograde (nucleus to organelle) and retrograde (organelle to nucleus) signaling pathways.^16–24^ However, fundamental challenges remain to be addressed, such as whether plant PCD requires communication between the two organelles and what is the nature of the signaling molecule(s) transduced between them.^4^

Our previous studies have shown that *mosaic death 1* (*mod1*) in *Arabidopsis thaliana* is a cell death mutant,^25^ which presents an elevated ROS level.^26^ Unlike the lesion mimic mutants, such as *accelerated cell death 2* (*acd2*)^27^ and *lesion simulating disease 1* (*lsd1*),^28^ which exhibit runaway cell death lesions, *mod1* shows a mosaic pattern of cell death in the leaf midvein and shoot meristem.^25^ The *MOD1* gene encodes an enoyl-acyl carrier protein (ACP) reductase (ENR), a subunit of fatty acid synthase that catalyzes the *de novo* biosynthesis of fatty acids in plastids; its deficiency leads to PCD.^25^ Genetic studies on the suppressors of *mod1* (*soms*) revealed that ROS accumulation and abnormal morphological phenotypes of *mod1* plants could be rescued by mutations affecting the activity of mETC complex I (NADH dehydrogenase/NADH oxidase), and that the ROS generated from mitochondrial ETC, rather than plasma membrane NADPH oxidases, plays a crucial role in the *mod1*-triggered PCD.^26^ The discovery that the abnormalities in *mod1* caused by the deficiency in fatty acid biosynthesis in the chloroplast can be restored by the decrease of mETC complex I activity suggests a signal produced in the chloroplast that ultimately induces ROS production by ETC in the mitochondrion. However, the identity of this signal and whether the signaling involves a direct communication with the mitochondrion are both unknown.^29^

To identify the signal transported from chloroplasts to induce ROS production in mitochondria, we now describe a screen for new *mod1* suppressors derived from ethyl methanesulfonate (EMS) mutagenized *Arabidopsis* M_2_ seeds. This has identified three key components in a direct Chloroplast-To-Mitochondrion (CTM) communication. In-depth studies on these genes reveal that the accumulation of MOD1 substrate NADH and its indirect transport from the chloroplast to the cytosol and then to the mitochondrion through the malate shuttle are responsible for ROS induction in mETC. This pathway is essential for redox homeostasis and plant growth regulation under different photoperiod conditions. We further demonstrate the existence of a conserved pathway from the cytosol to the mitochondrion that regulates ROS and cell death in HeLa cells. These findings identify a long suspected communication pathway from chloroplasts to mitochondria in plants and its conserved cytosol-mitochondrion ROS- and cell death-inducing mechanism in animals.

## Results

### Screening for *mod1* suppressors altered in the Chloroplast-To-Mitochondrion communication

**Fig. 1:**
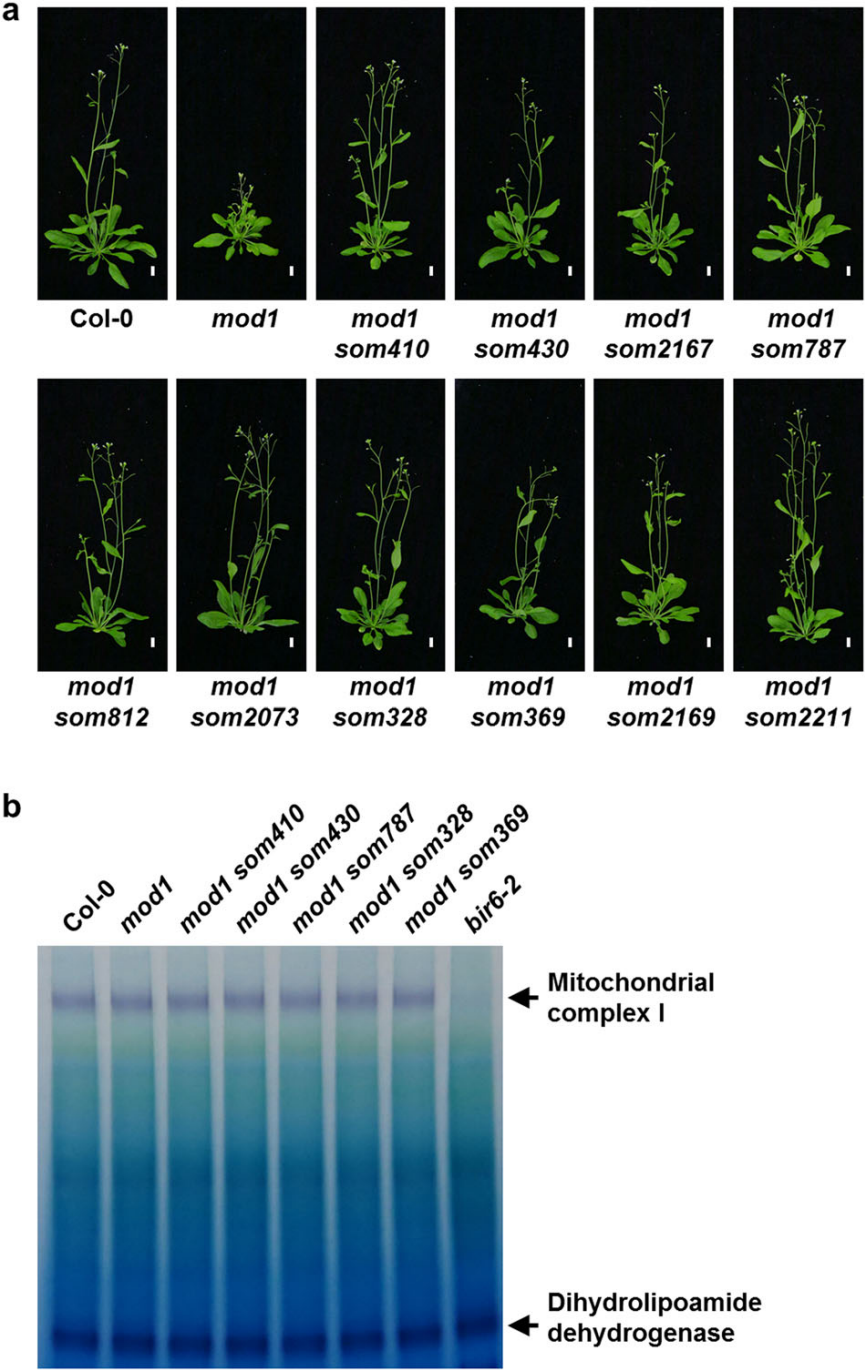
Screening for *mod1* suppressors that do not affect mitochondrial complex I activities. **a** Comparison of phenotypes of *som* mutants with the wild type and *mod1* plants at 30 days after germination (DAG). Scale bars, 1 cm. **b** In-gel assay of NADH oxidase activity with *bir6-2* as a control. The dihydrolipoamide dehydrogenase activity was used as a loading control

In the previous study with a T-DNA insertion mutant library, we had screened and identified many *soms*, most of which were mETC complex I-deficient mutants.^26^ To identify the components of the CTM communication, we generated an EMS mutagenized mutant library with more than 100,000 M_1_ plants and screened for new *soms* that are not the result of a deficiency in mETC complex I. Among 1073 *soms* identified in screening the EMS mutagenized mutant library, only 95 *soms* (8.9%) did not affect mETC complex I activity, of which we analyzed 10 *soms* in-depth in this study (Fig. 1).

### *SOM410* encodes a plastid-localized NAD-dependent malate dehydrogenase

**Fig. 2:**
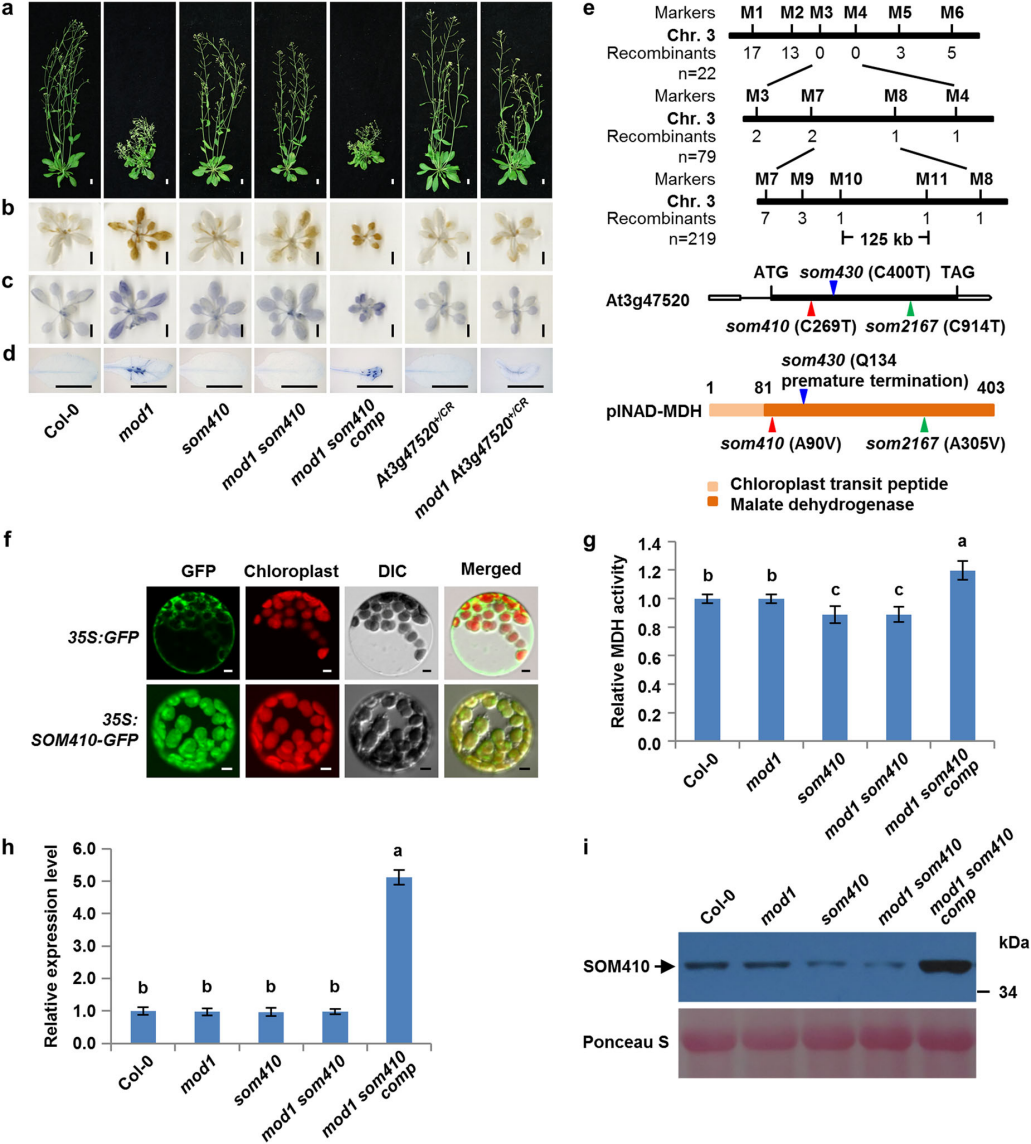
*SOM410* encodes and functions as a plastidial NAD-dependent malate dehydrogenase. **a** Phenotypes of Col-0, *mod1*, *som410*, *mod1 som410*, *mod1 som410 comp* (*mod1 som410* complemented line), *At3g47520*^*+/CR*^, and *mod1 At3g47520*^*+/CR*^ at 35 DAG. *At3g47520*^*+/CR*^ and *mod1 At3g47520*^*+/CR*^ were generated by CRISPR/Cas9 mutagenesis. Scale bars, 1 cm. **b** DAB-stained seedlings, showing the H_2_O_2_ levels. Scale bars, 1 cm. (**c**) NBT-stained seedlings, showing O_2_^−^ levels. Scale bars, 1 cm. **d** Trypan blue-stained leaves. Scale bars, 1 cm. **e** Map-based cloning of *SOM410* (top). The mutation sites in *som410*, *som430*, and *som2167* DNA (middle) and protein (bottom) sequences are indicated with red, blue and green triangles, respectively. **f** Subcellular localization of SOM410-GFP in *Arabidopsis* mesophyll protoplasts of *35S:SOM410-GFP* transgenic plants. Scale bars, 5 μm. **g** Comparison of total cellular MDH activities in the indicated plants. Values are means ± SD (*n* = 6), and different letters at top of each column indicate a significant difference at *P* < 0.05 determined by Tukey’s HSD test. **h** Transcript levels of *SOM410* in the indicated plants, revealed by qRT-PCR using *Actin* as reference. Values are means ± SD (*n* = 3), and different letters above each column indicate a significant difference at *P* < 0.05 determined by Tukey’s HSD test. **i** Protein levels of SOM410 in the indicated plants, detected by immunoblotting with anti-SOM410 polyclonal antibodies

To understand the nature and functions of the genes mutated in these *soms*, we systematically mapped and cloned these genes. Among the 10 *soms*, the homozygous recessive suppressor *som410* could fully suppress the *mod1* morphological and biochemical phenotypes including plant height, cell death, and levels of H_2_O_2_ and O_2_^−^, showing a similar phenotype to that of the wild type (Fig. 2a–d). To clone *SOM410*, we took a map-based cloning approach and located the *SOM410* gene in a 125-kb region on chromosome 3 (Fig. 2e). Through whole-genome resequencing of *mod1 som410*, we identified a C269T point mutation in *At3g47520* gene in the mapped region (Fig. 2e). This mutation leads to an A90V amino acid substitution in the protein sequence. To verify that the suppression of the *mod1* phenotypes was truly caused by this point mutation in *At3g47520*, the *mod1 som410* mutant was complemented by a 3.1-kb genomic DNA fragment containing the *At3g47520* gene. All the transformants (*P*_*SOM410*_*:SOM410/mod1 som410*, designated as *mod1 som410 comp*) resumed the *mod1* phenotypes (Fig. 2a–d), demonstrating that the suppression of the *mod1* phenotypes is caused by the mutation in *At3g47520*. In addition, we identified another two independent suppressors, *som430*^*+/-*^ and *som2167*, which were mapped to the similar region as *som410* and could partially or fully suppress the cell death in *mod1*. Whole-genome resequencing results showed that both of them have mutations in *At3g47520*. The *som430*^*+/-*^ mutant has a C400T point mutation leading to a premature translation termination (Fig. 2e and Supplementary information, Figure S1a-e), whereas *som2167* carries a C914T point mutation resulting in an A305V amino acid change (Fig. 2e and Supplementary information, Figure S2a and S2b). Therefore, these results further confirm that the mutations in *At3g47520* are responsible for the suppression of the *mod1* phenotypes in *mod1 som410*, *mod1 som430*^*+/-*^, and *mod1 som2167*.

The *At3g47520* (*SOM410*, *SOM430*, or *SOM2167*) locus encodes a plastidial NAD-dependent malate dehydrogenase (plNAD-MDH).^30–32^ Phylogenic analysis showed that the MDH domain is highly conserved across different plant species but the chloroplast transit peptide domain is less conserved (Supplementary information, Figure S3). To confirm the function of SOM410, we first examined its subcellular localization. The SOM410 protein was clearly localized in the chloroplast of the *35S:SOM410-GFP* transgenic plants, which is in accord with its annotation and previous reports^30–32^ (Fig. 2f). We then measured and compared the total MDH activities among the relevant genotypes. The MDH activities were similar between the wild type and *mod1*, but they were significantly impaired in both *som410* and *mod1 som410* compared to the wild type and *mod1* (Fig. 2g). As expected, the MDH activity was restored in all the complemented lines (*mod1 som410 comp*) compared to *mod1 som410*. We observed similar results among *som430*^*+/-*^, *mod1 som430*^*+/-*^, and complemented lines (*mod1 som430*^*+/-*^ *comp1* and *2*) (Supplementary information, Figure S1i). These results confirm that *SOM410* encodes a plastidial malate dehydrogenase.

To understand why the point mutation in *som410* affects the MDH activity, we examined the expression levels of *SOM410* by quantitative real-time PCR (qRT-PCR). We found no significant difference between wild type, *mod1*, *som410*, and *mod1 som410*, but in the complemented line the *SOM410* transcript level was significantly increased (Fig. 2h). Moreover, we found that the SOM410 protein levels were obviously decreased in *som410* and *mod1 som410* compared with the wild type but strikingly increased in the complemented line (Fig. 2i), which is in line with the MDH activities in the corresponding lines. Taken together, these results suggest that the point mutation of *som410* could reduce the stability of the SOM410 protein, which in turn leads to the deficiency of the MDH activity in chloroplasts.

To further understand the function of *SOM410*, we also attempted to generate *At3g47520* loss-of-function mutants using the CRISPR/Cas9 system. We did not obtain any homozygous loss-of-function mutants of *At3g47520*, suggesting that the null mutation of *SOM410* is lethal, which accords with previous reports that the T-DNA insertion mutant of plNAD-MDH was embryonic lethal at the globular-to-heart transition stage.^30,32^ However, we obtained a heterozygous mutant (*At3g47520*^*+/CR*^) with a T insertion at 178 bp downstream of ATG start codon, and this mutant partially rescued *mod1* phenotypes (Fig. 2a–d and Supplementary information, Figure S2c). Similarly, the homozygous mutation of *SOM430*, which resulted in a premature translation termination of the protein, is also embryonic lethal. The segregation of *mod1 som430*^*+/-*^ resulted in 303 suppressed to 160 non-suppressed plants in the self-pollinated progeny, fitting the theoretical segregation ratio of 2:1 (*P*-value = 0.58 by chi-square test). Moreover, overexpression of *At3g47520* in *mod1 som430* ^*+/-*^ resumed the *mod1* phenotypes (Supplementary information, Figure S1a-d and S1f-h). Therefore, chloroplastic NAD-MDH is indispensable in plants, and partial loss of function of chloroplastic NAD-MDH in *som410*, *som430*^*+/-*^, and *som2167* is sufficient for the suppression of *mod1* phenotypes.

### *SOM787* encodes a chloroplastic dicarboxylate transporter

**Fig. 3:**
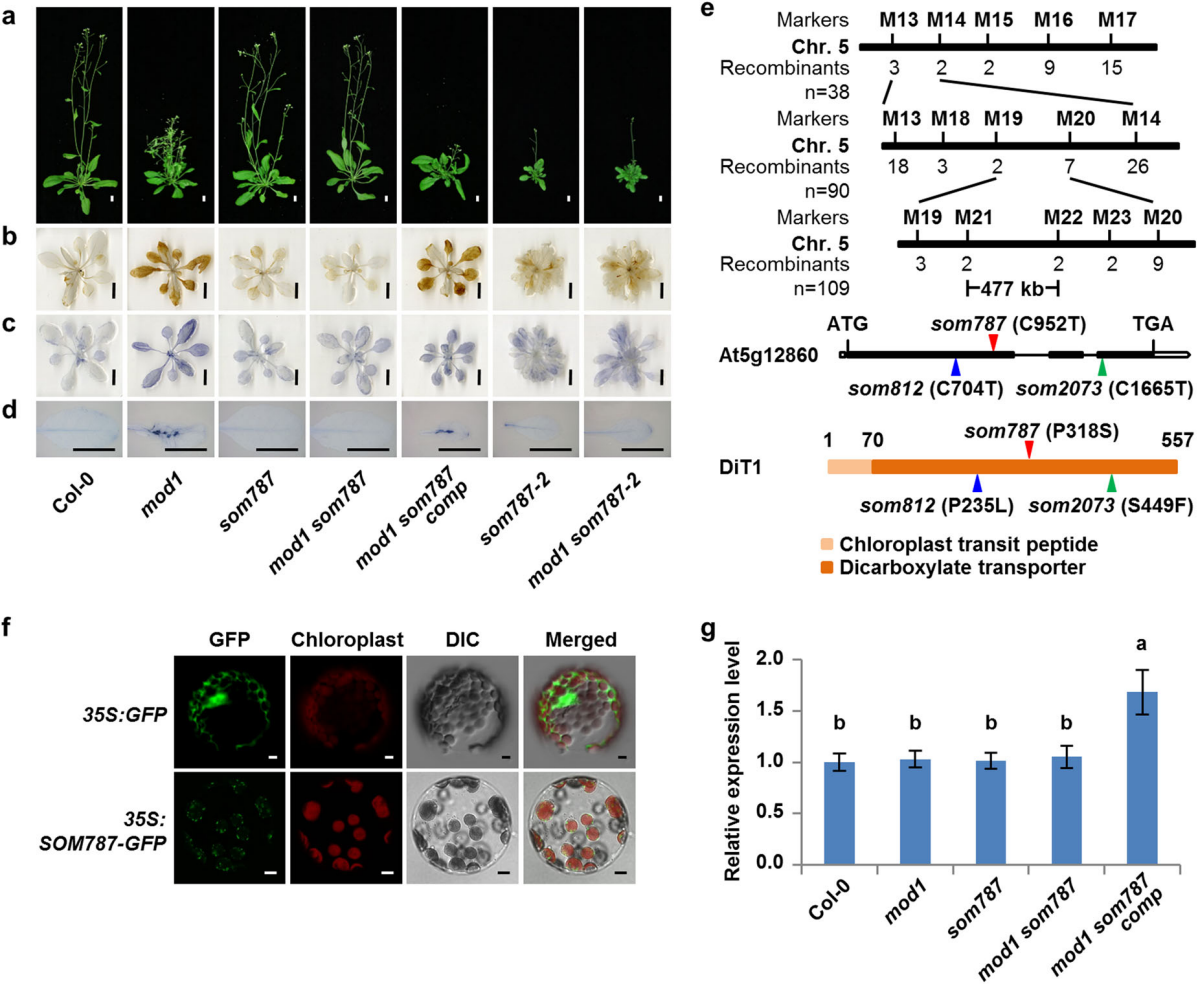
Cloning and characterization of *SOM787*. **a** Phenotypes of Col-0, *mod1*, *som787*, *mod1 som787*, *mod1 som787 comp* (*mod1 som787* complemented line), *som787-2* (N877710), and *mod1 som787-2* at 32 DAG. Scale bars, 1 cm. **b** DAB-stained seedlings. Scale bars, 1 cm. **c** NBT-stained seedlings. Scale bars, 1 cm. **d** Trypan blue-stained leaves. Scale bars, 1 cm. **e** Map-based cloning of *SOM787* (top). The mutation sites in *som787*, *som812*, and *som2073* DNA (middle) and protein (bottom) sequences are the indicated with red, blue and green triangles, respectively. **f** Subcellular localization of SOM787-GFP in *Arabidopsis* mesophyll protoplasts of *35S:SOM787-GFP* transgenic plants. Scale bars, 5 μm. **g** Transcript levels of *SOM787* revealed by qRT-PCR using *Actin* as reference. Values are means ± SD (*n* = 3), and different letters at top of each column indicate a significant difference at *P* < 0.05 determined by Tukey’s HSD test.

The suppressor *som787* is another type of recessive mutant that fully rescued the *mod1* phenotypes (Fig. 3a–d). We mapped the *som787* mutation to a 477-kb region on chromosome 5. Through whole-genome resequencing, we identified a C952T point mutation in *At5g12860* gene in the mapped region (Fig. 3e). This mutation results in a P318S amino acid substitution in protein sequence. The *mod1 som787* mutant was complemented by a 4.2−kb genomic DNA fragment containing the *At5g12860* gene, and all the transgenic plants (*P*_*SOM787*_*:SOM787/mod1 som787*, designated as *mod1 som787 comp*) were restored to the *mod1* phenotypes (Fig. 3a–d). We also identified another two independent *som* mutants, *som812* and *som2073*. Both *som812* and *som2073* were mapped to the same region as *som787*. *som812* contained a C704T point mutation in *At5g12860* leading to a P235L amino acid substitution (Fig. 3e and Supplementary information, Figure S4a and b), whereas *som2073* carried a C1665T point mutation in *At5g12860* leading to an S449F amino acid substitution (Fig. 3e and Supplementary information, Figure S4a and c). Therefore, the mutations in *At5g12860* are responsible for the suppression of *mod1* phenotypes. We additionally obtained a T-DNA insertion mutant of *At5g12860*, *som787-2* (N877710), which contains a T-DNA insertion at 531 bp downstream of ATG start codon. The transcript level of *At5g12860* is significantly decreased in *som787-2* (Supplementary information, Figure S5). In contrast to the observation that *som787* had no obvious abnormal phenotypes compared with Col-0, *som787-2* plants were smaller in size, indicating that *som787*, *som812* and *som2073* are leaky mutants. The *mod1 som787-2* double mutant showed similar morphology to *som787-2*, and was rescued for the *mod1* phenotypes of cell death and accumulation of H_2_O_2_ and O_2_^−^ (Fig. 3a–d).

*SOM787* is allelic to a previously reported gene, *DICARBOXYLATE TRANSPORTER 1* (*DiT1*), which encodes a chloroplastic dicarboxylate transporter that functions as the malate/oxaloacetate (OAA) transporter or as the 2-oxoglutarate (2-OG)/malate transporter to export malate out of the chloroplast.^33,34^ Consistent with its transporting functions, we found that SOM787 is specifically localized in the chloroplast envelope membrane (Fig. 3f). The expression level of *SOM787* is not affected in *som787*, indicating that the proline at 318 position (P318) is crucial for its normal function (Fig. 3g). Phylogenic analysis revealed that P318 and the dicarboxylate transporter domain of SOM787 are highly conserved across different plant species while the chloroplast transit peptide domain is less conserved (Supplementary information, Figure S6). Two paralogues of *SOM787*, *DiT2.1* and *DiT2.2*, are found in *Arabidopsis thaliana* (Supplementary information, Figure S7a), of which only DiT2.1 has been reported as a glutamate/malate translocator able to transport malate into chloroplasts.^34^ We therefore crossed *mod1* with two T-DNA insertion alleles of these two genes (*dit2.1-1*, *dit2.1-2*, *dit2.2-1*, and *dit2.2-2*). Morphological analysis showed that both *mod1 dit2.1-1* and *mod1 dit2.1-2* had severe growth phenotypes (Supplementary information, Figure S7b), similar to *dit2.1-1* and *dit2.1-2* as previously reported.^34,35^ Furthermore, neither *dit2.2-1* nor *dit2.2-2* could rescue the *mod1* phenotypes (Supplementary information, Figure S7c). These results suggest that suppressing *mod1* phenotypes by *DiT1* mutations is closely related to the function of DiT1 as a transporter to export malate from chloroplasts.

### *SOM328* encodes a mitochondrial malate dehydrogenase

**Fig. 4:**
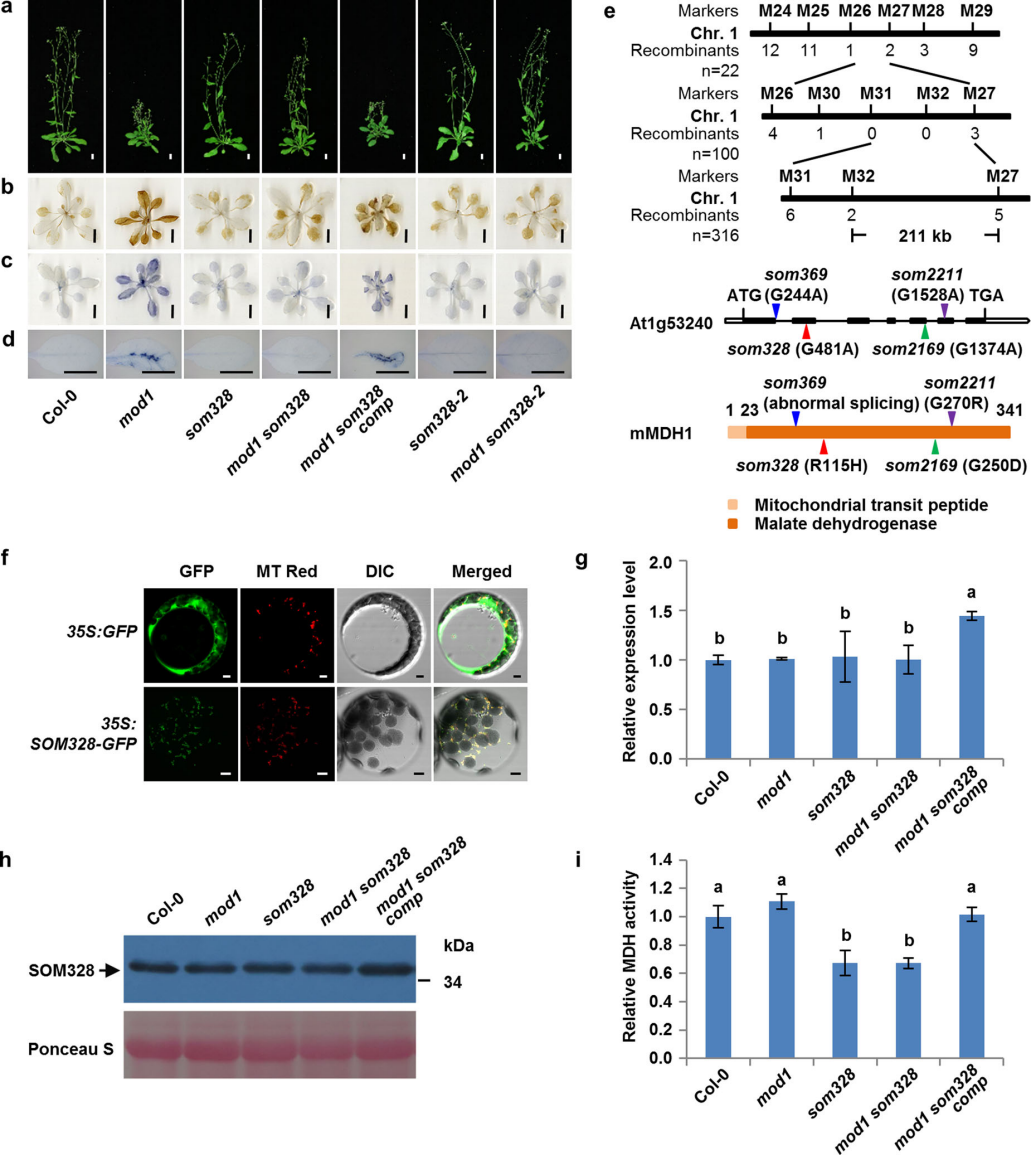
Identification of *SOM328* as a mitochondrial malate dehydrogenase, which is required for the generation of ROS and cell death in *mod1*. **a** Phenotypes of Col-0, *mod1*, *som328*, *mod1 som328*, *mod1 som328 comp* (*mod1 som328* complemented line), *som328-2* (N362639), and *mod1 som328-2* at 32 DAG. Scale bars, 1 cm. **b** DAB-stained seedlings. Scale bars, 1 cm. **c** NBT-stained seedlings. Scale bars, 1 cm. **d** Trypan blue-stained leaves. Scale bars, 1 cm. **e** Map-based cloning of *SOM328* (top). The mutation sites in *som328*, *som369*, *som2169*, and *som2211* DNA (middle) and protein (bottom) sequences are indicated with red, blue, green and purple triangles, respectively. **f** Subcellular localization of SOM328-GFP in *Arabidopsis* mesophyll protoplasts of *35S:SOM328-GFP* transgenic plants. Scale bars, 5 μm. **g** Transcript levels of *SOM328* revealed by qRT-PCR with *Actin* as reference. Values are means ± SD (*n* = 3), and different letters at top of each column indicate a significant difference at *P* < 0.05 determined by Tukey’s HSD test. **h** Protein levels of SOM328 in the indicated plants, detected by immunoblotting with anti-SOM328 polyclonal antibodies. **i** Comparison of total cellular MDH activities in the indicated plants. Values are means ± SD (*n* = 6), and different letters at top of each column indicate a significant difference at *P* < 0.05 determined by Tukey’s HSD test

The suppressor *som328* represents the third type of recessive mutants that rescued the *mod1* phenotypes (Fig. 4a–d). We mapped *som328* to a 211-kb region on chromosome 1. By whole-genome resequencing, we identified a G481A point mutation in *At1g53240* gene in this region. This mutation leads to an R115H amino acid change in protein sequence (Fig. 4e). The *mod1 som328* mutant was complemented by a 4.2-kb genomic DNA fragment containing *At1g53240* (*P*_*SOM328*_*:SOM328/mod1 som328*, designated as *mod1 som328 comp*) and we found that the *mod1* phenotypes were fully restored (Fig. 4a–d). Consistent with this result, we also found three additional suppressors, *som369*, *som2169*, and *som2211*, which all contained mutations in the *At1g53240* gene. The *som369* mutant has a G244A point mutation at the first nucleotide of intron 1 leading to abnormal splicing with dramatically decreased expression level and showed significantly impaired total MDH activity (Fig. 4e and Supplementary information, Figure S8); *som2169* has a G1374A point mutation leading to the G250D amino acid change (Fig. 4e and Supplementary information, Figure S9a and b); and *som2211* has a G1528A point mutation leading to the G270R amino acid change (Fig. 4e and Supplementary information, Figure S9a and c). These results demonstrate that the mutations in *At1g53240* are responsible for the suppression of the *mod1* phenotypes. In addition, we found that a T-DNA insertion mutant of *At1g53240*, *som328-2* (N362639^36^), fully rescued the *mod1* phenotypes (Fig. 4a–d).

*SOM328* is allelic to the previously reported gene, *MITOCHONDRIAL MALATE DEHYDROGENASE 1* (*mMDH1*).^36^ Phylogenic analysis revealed that the malate dehydrogenase domain of SOM328 is highly conserved not only in different plant species but also in *Caenorhabditis elegans*, *Drosophila melanogaster*, and mammalian species, while the mitochondrial transit peptide domain is diverse (Supplementary information, Figure S10). To confirm the localization of the SOM328 protein in mitochondria, we examined its subcellular localization by stably expressing *35S:SOM328-GFP* in the wild type (Col-0) and found that the GFP florescent signal co-localized with the mitochondria-specific marker, MitoTracker Red (Fig. 4f). Furthermore, we found that the point mutation in *som328* did not affect its RNA level or the protein level, suggesting that the arginine at the 115 position may affect the mMDH enzyme activity (Fig. 4g, h). We therefore compared the total MDH activities in all relevant lines. No differences have been observed among the wild type, *mod1* and complemented line (*mod1 som328 comp*). However, *som328* or *mod1 som328* showed significant decreased total MDH activities (Fig. 4i). Taken together, these results demonstrate that the mitochondrial malate dehydrogenase plays an essential role in causing *mod1* phenotypes.

### Malate shuttle is responsible for the organelle communication in PCD of *mod1*

**Fig. 5:**
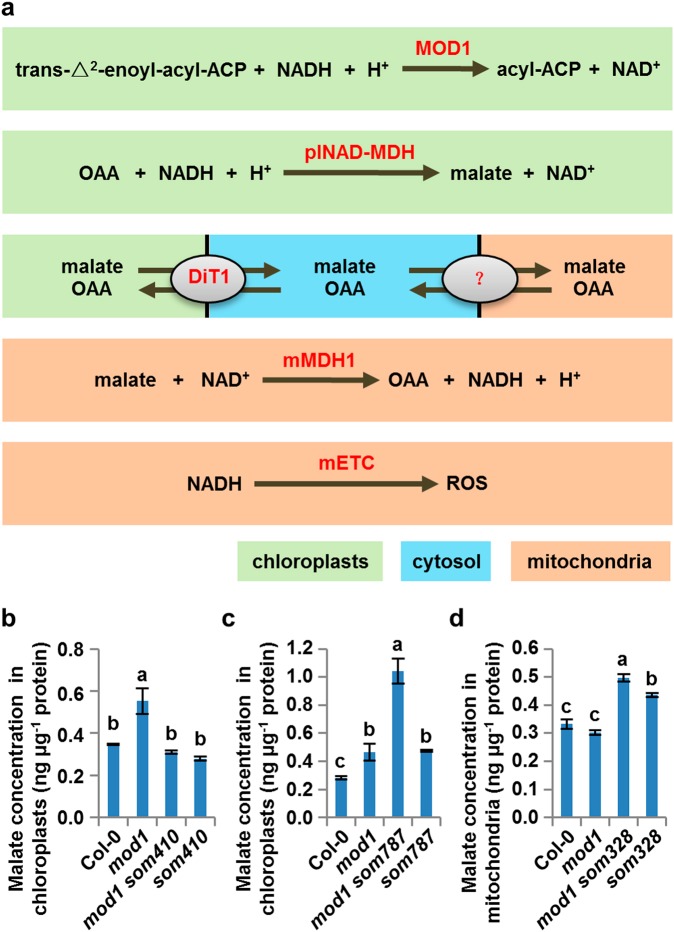
Malate shuttle carries the Chloroplast-To-Mitochondrion signal in *mod1*. **a** Schematics of the locations and functions of SOMs. plNAD-MDH (plastidial NAD-dependent malate dehydrogenase) is encoded by *SOM410*, *SOM430* or *SOM2167*; DiT1 (chloroplastic dicarboxylate transporter 1) by *SOM787*, *SOM812* or *SOM2073*; and mMDH1 (mitochondrial malate dehydrogenase 1) by *SOM328*, *SOM369*, *SOM2169*, or *SOM2211*. **b** Changes of malate concentration in chloroplasts isolated from Col-0, *mod1*, *mod1 som410*, and *som410*. **b**–**d** Values are means ± SD (*n* = 3), and different letters at top of each column indicate a significant difference at *P* < 0.05 determined by Tukey’s HSD test. **c** Changes of malate concentration in chloroplasts isolated from Col-0, *mod1*, *mod1 som787*, and *som787*. **d** Changes of malate concentration in mitochondria isolated from Col-0, *mod1*, *mod1 som328* and *som328*

The genetic and functional confirmation of the *SOMs* identified above allows us to draw a coherent picture of the CTM communication in *mod1*-triggered PCD (Fig. 5a). MOD1, an enoyl-ACP reductase, utilizes NADH to reduce the double bond in the fatty acyl group, and deficiency in MOD1 will favorably lead to the accumulation of the reducing equivalent NADH in the chloroplast and trigger plNAD-MDH to reduce OAA to malate. The over-produced malate in the chloroplast is then exported into the cytosol by DiT1, which is localized in the chloroplast inner envelope membrane. The cytosolic malate is in turn transported into the mitochondrion and oxidized to OAA by mMDH1, coupled to the generation of NADH. NADH then serves as the electron donor for the mETC to generate ROS. Therefore, it is likely that direct transport of malate from chloroplast to mitochondrion through the malate shuttle provides the chemical communication pathway by which the excess reducing equivalent in the chloroplast is conveyed to the mitochondrion, leading to the generation of ROS and PCD in *mod1* plants.

If the malate shuttle is truly carrying the CTM communication signal of organelle communication, then the malate concentrations in the *mod1* mutant and various suppressors should show corresponding changes in chloroplasts and mitochondria. We therefore measured and compared the malate concentrations in *mod1* and *mod1 som410*, *mod1 som787*, or *mod1 som328* (Fig. 5). Compared with *mod1*, the malate concentration in the chloroplasts of *mod1 som410* was significantly decreased and restored to the wild-type level, reflecting the deficiency in conversion from OAA to malate as a result of the *SOM410* mutation (Fig. 5b). In contrast, the malate concentration in the chloroplasts of *mod1 som787* was significantly increased and much higher than that in the chloroplasts of *mod1*, because export of malate from the chloroplast was impaired (Fig. 5c). In mitochondria, the malate concentration in *mod1 som328* is significantly elevated compared with *mod1*, because of the deficient conversion from malate to OAA as a result of the *SOM328* mutation (Fig. 5d). Together, these data further demonstrate that the malate shuttle is responsible for the CTM communication from the chloroplast, the energy-generating organelle, to the mitochondrion, the energy-transforming organelle, to regulate ROS formation.

To investigate whether this CTM communication is involved in the cell death pathway of the lesion mimic mutants, we generated the double mutants by crossing different *soms* including *som410*, *som787*, *som328*, and *som3* (an mETC complex I deficient mutant)^26^ with *acd2*^27^ or *lsd1* (N542687),^37^ two typical lesion mimic mutants. We found that the cell death phenotypes in *acd2* and *lsd1* are not suppressed by *soms* (Supplementary information, Figure S11). These results indicate that neither the CTM communication nor mETC-generated ROS are involved in the cell death regulation in* acd2* and *lsd1*, and PCD triggered by *mod1* is controlled by mechanisms different from that in *acd2* and *lsd1*.

### ROS by CTM communication affects plant growth under different photoperiod conditions

**Fig. 6:**
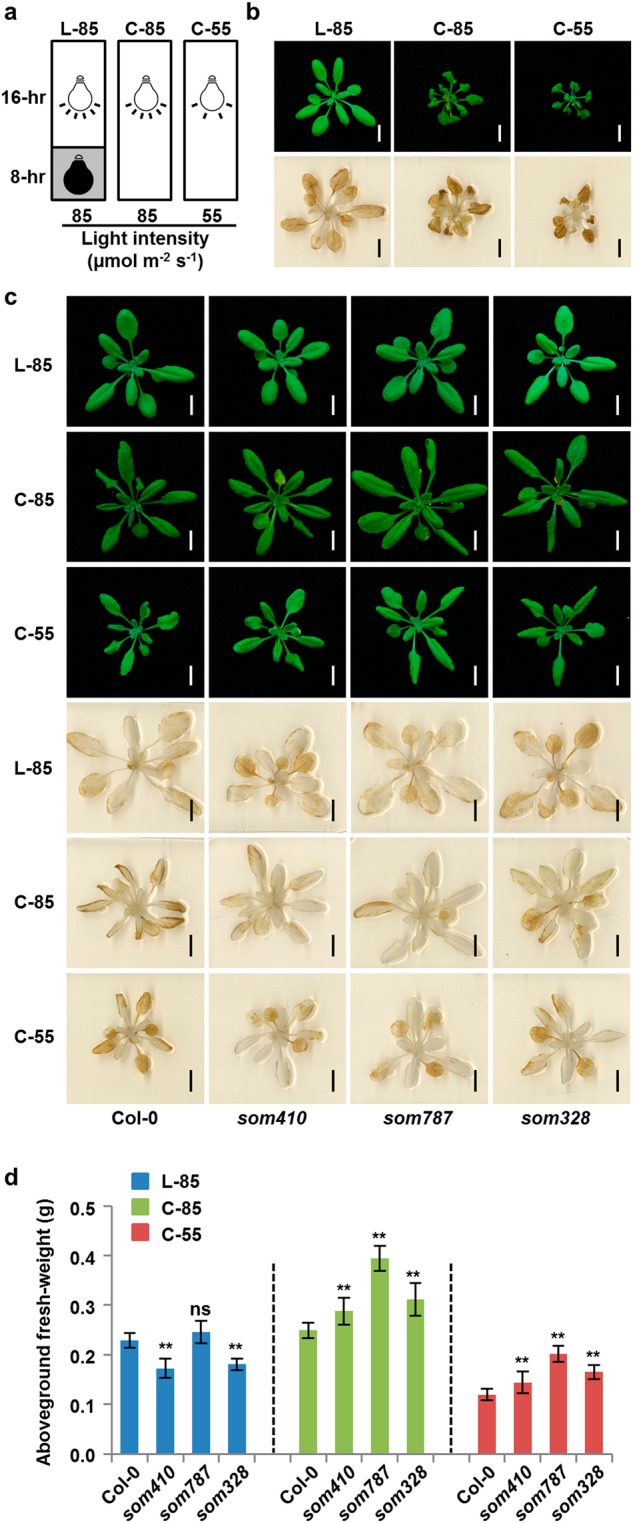
ROS generated through the malate shuttle affects plant growth under different photoperiod conditions. **a** Schematics of different growth conditions. L-85, long-day condition (16-hr light/8-hr dark) with a light intensity of 85 µmol m^−2^ s^−1^; C-85 or C-55, continuous illumination conditions (24-hr light) with a light intensity of 85 µmol m^−2^ s^−1^ or 55 µmol m^−2^ s^−1^. **b** Phenotypes and DAB staining of *mod1* grown under different illumination conditions in **a** at 22 DAG. Scale bars, 1 cm. **c** Phenotypes and DAB staining of Col-0, *som410*, *som787*, and *som328* grown under different illumination conditions in **a** at 22 DAG. Scale bars, 1 cm. **d** Aboveground fresh weight of Col-0, *som410*, *som787*, and *som328* under different illumination conditions in **a** at 22 DAG. Values are means ± SD (*n* = 10). The asterisks represent significant difference between mutant and Col-0 grown under the same condition determined by Student’s t test. ns, no significant difference; *P < 0.05; **P < 0.01

Photoperiod is a major environmental factor controlling plant growth and development. Photoperiod could affect a plant’s redox state and is considered as a crucial factor in the regulation of H_2_O_2_-induced cell death.^38^ However, the regulatory mechanism remains to be determined. The *mod1* mutant was originally identified under continuous light condition, leading us to compare the *mod1* phenotype under long-day condition (16-hr light/8-hr dark) and continuous light conditions (24-hr light). This revealed that both the PCD phenotype and ROS accumulation in *mod1* become more severe under continuous light conditions (Fig. 6a, b). Moreover, the phenotypes of *mod1* were still very severe under continuous light condition when the light intensity was decreased from 85 μmol m^−2^ s^−1^ to 55 μmol m^−2^ s^−1^ (Fig. 6b), indicating that the photoperiod condition affects the *mod1* phenotypes and the ROS generation. Taken together with the findings that the interruption of the malate shuttle could suppress PCD and ROS accumulation in *mod1*, it follows that the CTM communication is involved in ROS accumulation under the continuous light conditions. We therefore grew the wild type and three suppressor mutants, *som410*, *som787* and *som328*, under three different conditions (long-day condition with 85 μmol m^−2^ s^−1^ and continuous light conditions with either 85 or 55 μmol m^−2^ s^−1^). We found that ROS levels in Col-0 were higher under the continuous light conditions than long-day condition (Fig. 6c), indicating that the continuous light could induce ROS accumulation. More importantly, the ROS levels of three suppressor mutants were similar to that of Col-0 under the long-day condition, but lower than that of Col-0 under the continuous light conditions (Fig. 6c). This indicates that CTM communication plays an important role in regulating ROS accumulation under the continuous light conditions.

Considering that the CTM communication plays an important role in regulating plant growth, we therefore compared plant size and aboveground fresh weight of *som410*, *som787* and *som328* with the wild type. We found that under both continuous light conditions plant size and fresh weight of all three suppressor mutants were remarkably larger (Fig. 6d), which accords with the decreased ROS levels in suppressor mutants (Fig. 6c). These results suggest that CTM communication plays an important role in regulating redox homeostasis and thereby affecting plant growth in response to different photoperiod conditions and that the impairment in the CTM communication could reduce the oxidative damages caused by continuous light.

### Malate induces ROS generation and cell death in HeLa cells

**Fig. 7:**
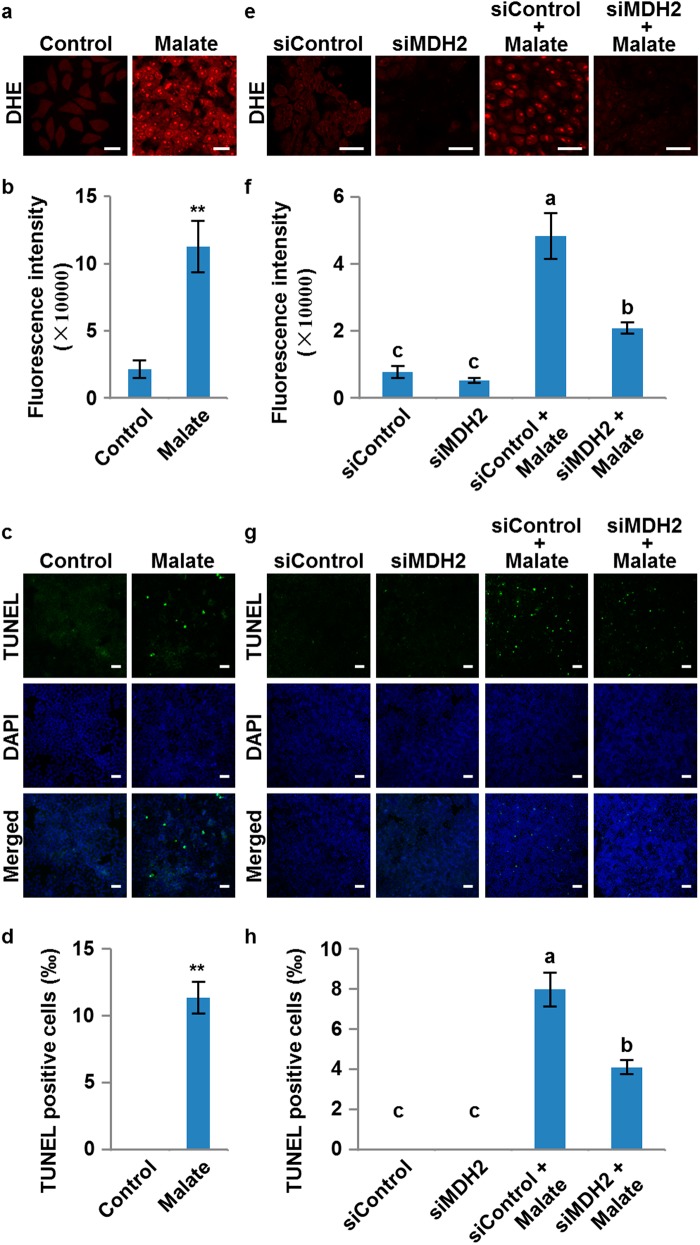
ROS induction and cell death triggered by malate in HeLa cells. **a** ROS levels revealed by dihydroethidium (DHE) staining in HeLa cells with or without malate treatment. Scale bars, 25 μm. **b** Quantitation of data in **a**. Values are means ± SD (*n* = 3) based on 3 independent experiments. The asterisks represent significant difference determined by Student’s *t* test. **P < 0.01. **c** Cell death levels revealed by TUNEL staining in HeLa cells with or without malate treatment. Scale bars, 50 μm. **d** Quantitation of data in **c**. Values are means ± SD (*n* = 3) based on three independent experiments. The asterisks represent significant difference determined by Student’s *t* test. **P < 0.01. **e** ROS levels revealed by DHE staining in control (siControl) and *MDH2* knockdown HeLa cells (siMDH2) with or without malate treatment. Scale bars, 25 μm. **f** Quantitation of data in **e**. Values are means ± SD (*n* = 3) based on three independent experiments, and different letters at top of each column indicate a significant difference at *P* < 0.05 determined by Tukey’s HSD test. **g** Cell death levels revealed by TUNEL staining in control (siControl) and *MDH2* knockdown HeLa cells (siMDH2) with or without malate treatment. Scale bars, 100 μm. **h** Quantitation of data in **g**. Values are means ± SD (*n* = 3) based on three independent experiments, and different letters at top of each column indicate a significant difference at *P* < 0.05 determined by Tukey’s HSD test

It has been reported that animals and plants show some similar morphological and biochemical features of PCD.^3^ However, whether they share the same mechanism in regulating ROS generation and triggering PCD is still an open fundamental biological question. Since malate and NADH are ubiquitous primary metabolic molecules in all cells and the malate dehydrogenase domain and the function of mMDH is highly conserved across plants and animals, we asked whether the PCD pathway regulated by malate is conserved between plants and animals. To illustrate this, we used HeLa cells as a working system. Based on the report that the abundance of malate in different tissues of mice varies from ~60 to 386 ng/mg^39^ and considering the uptake efficiency, we treated the cells with 50 mM malate for 24 h and then measured the levels of ROS and cell death. The malate-treated cells showed a significantly increased ROS level detected by dihydroethidium (DHE) staining (Fig. 7a, b). Terminal deoxynucleotidyl transferase-mediated dUTP nick end labeling (TUNEL) showed that the cell death levels were also significantly elevated in malate-treated cells (Fig. 7c, d). This is consistent with a previous study in human keratinocyte cell lines, in which treatment with exogenous malate could significantly increase the production of mitochondrial superoxide and induce PCD through mitochondria-dependent pathways.^40^ These results indicate that the ROS accumulation and PCD induced by malate are conserved processes in both plants and animals. More importantly, we examined whether mMDH also plays a conserved role in this process in animals. We identified human *MDH2* gene, which is an ortholog of *Arabidopsis mMDH1* and encodes a mitochondrial MDH. We then generated the *MDH2* knockdown HeLa cells (siMDH2) by siRNA (Supplementary information, Figure S12). Remarkably, after treatment with malate for 24 h, both ROS and cell death levels were significantly decreased in siMDH2 cells compared with the control cells (siControl) (Fig. 7e–h), suggesting that MDH2 plays a vital role in regulating the malate-induced PCD pathway in human cells. Taken together, these results demonstrate that generation of ROS and PCD regulated by the malate shuttle are conserved processes in both plant and animal cells.

## Discussion

**Fig. 8:**
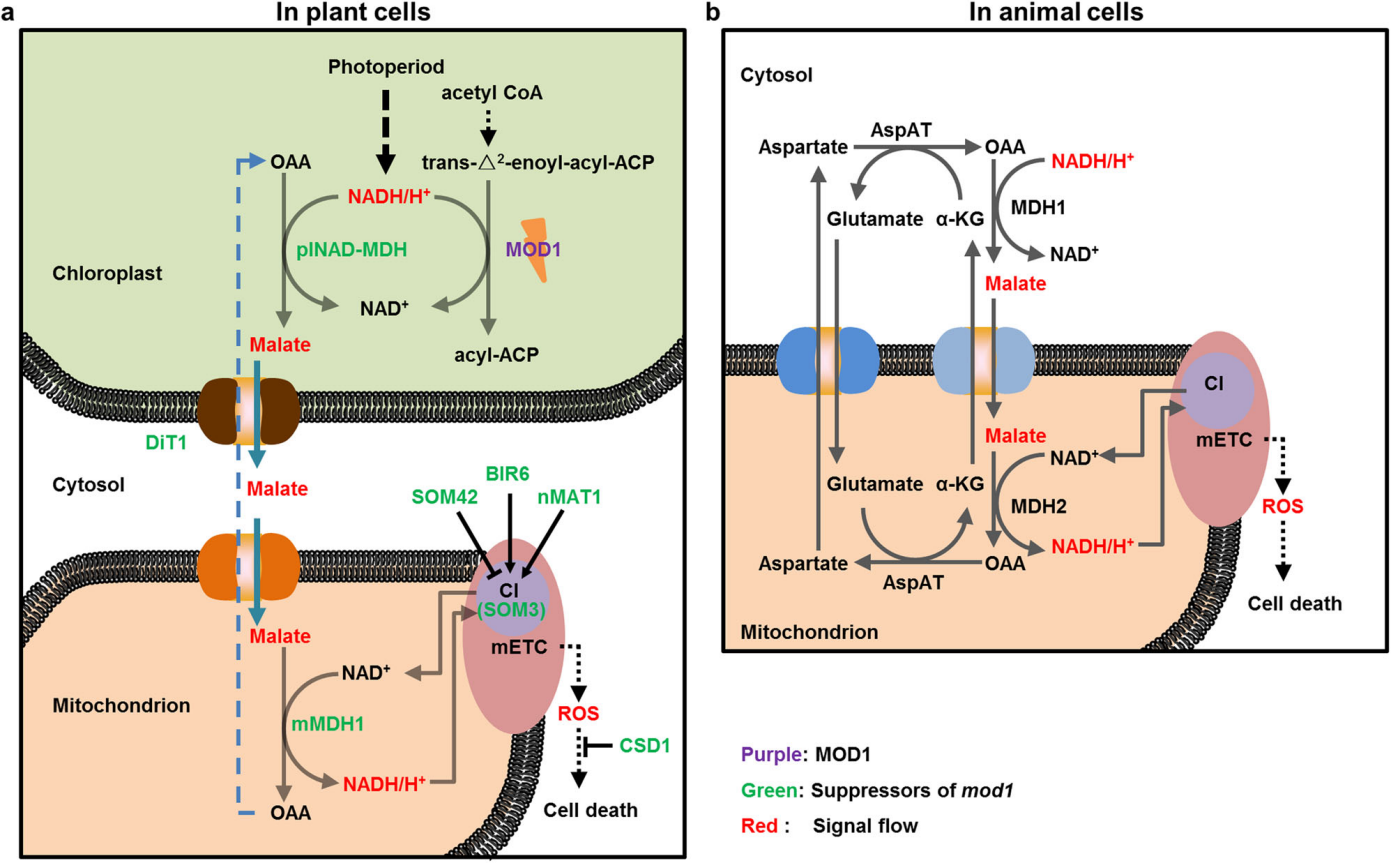
A proposed model of programmed cell death in plant and animal cells. **a** In plants, the deficiency of MOD1 leads to an increased level of NADH in the chloroplast, which drives OAA to be converted to malate by plNAD-MDH. Malate carrying the reducing equivalents is transported out of the chloroplast into the cytosol by DiT1, and then be transported into the mitochondrion by an unidentified transporter or transporters. In the mitochondrion, malate is converted to OAA by mMDH1, and simultaneously NADH is generated to provide electrons for mETC to induce the ROS formation and initiate the PCD process in the *mod1* cells. **b** In HeLa cells, exogenous malate could be taken into the cytosol, and then transported into the mitochondrion by the malate/α-ketoglutarate (α-KG) transporter. In the mitochondrion, malate is converted to OAA by MDH2, and simultaneously NADH is generated to provide electrons for mETC to induce the ROS formation and initiate the cell death process, which is highly conserved with the PCD in *mod1* plants

Communication between the nucleus and organelles in eukaryotic cells plays vital roles in responding to different environmental stresses and in coordinating compartmentally localized materials and signals to regulate cell growth and differentiation. The signaling pathways from the nucleus to organelles (anterograde signaling) and from organelles to the nucleus (retrograde signaling) have been well studied over the past several decades.^16–24^ In recent years, studies of the communication between organelles have revealed its indispensable roles in executing cellular activities for almost all the life processes, for example, the communication or interaction of the endoplasmic reticulum (ER) with the Golgi, mitochondrion, chloroplast, or lysosome.^41–45^ Compared with animal cells, a higher plant cell not only contains a rigid cell wall and a large vacuole, but also possesses a unique phototrophic energy-generating system in the chloroplast that produces bioenergy for nearly all living organisms. Therefore, it is naturally believed that communication must exist between the chloroplast, an organelle generating chemical energy, and the mitochondrion, an organelle transforming chemical energy, and that such communication plays an essential role in regulating plant growth and development. Recent reports suggest that there is a CTM communication, direct or indirect, in *mod1*-triggered PCD in *Arabidopsis thaliana*,^26,29^ but its nature and mechanism are still elusive. In this work, we demonstrate that in plants, the malate shuttle conveys a direct communication signal from the chloroplast to mitochondrion presumably via the cytosol (Fig. 8a). Moreover, we further demonstrate that the pathway functions to deliver reducing equivalents for mitochondrial ROS generation, providing a communication system which reports the cellular redox status and mediates cell death in animal cells (Fig. 8b).

It has been well established that the malate shuttle is a fundamental mechanism to regulate the redox homeostasis among different compartmentalised organelles of a eukaryotic cell by indirectly transporting reducing equivalents across membranes.^30,46–50^ *mod1* and *soms* provides us an ideal model system to investigate the mechanism underlying the communication between the chloroplast and mitochondrion. Under a long illumination condition, the reducing equivalent NADH appears to accumulate due to its reduced consumption resulting from a deficiency in the biosynthesis of fatty acids in *mod1* chloroplasts. The accumulated reducing equivalent drives the formation of malate from OAA catalyzed by plNAD-MDH, the plastidial NAD-dependent malate dehydrogenase (Fig. 8a). The malate formed is then exported into the cytosol by a plastid-specific transporter DiT1 and subsequently transported into mitochondria by an unknown transporter. In the mitochondrial matrix, the malate is oxidized to OAA by mMDH, together with generation of the reducing equivalent NADH, which is used as the electron donor to mETC for ATP production by oxidative phosphorylation but will lead to ROS production in cells with redox imbalance (Fig. 8a). Malate functions as an important compound in cell metabolism and a carrier of the reducing equivalents, and it is the malate shuttle that plays an essential role in maintaining the redox homeostasis required for organelles. The malate shuttle could trigger the mitochondrial ROS generation and activate the downstream PCD pathway in response to different conditions that affect redox homeostasis. In higher plants, this mechanism ensures the reducing equivalents generated in the chloroplast are exported and utilized for the growth and development, and this may explain why the null mutation in *plNAD-MDH* is lethal.

The photoperiod is considered as a crucial factor in the regulation of H_2_O_2_-induced cell death.^38^ The *Arabidopsis thaliana catalase 2* (*cat2*) mutant shows different levels of cell death under different photoperiod conditions due to the accumulation of H_2_O_2_.^38^ Hundreds of downstream genes show day length-dependent responses to intracellular H_2_O_2_ at transcript levels,^51^ and the H_2_O_2_-activated glutathione synthesis is influenced by the photoperiod in *Arabidopsis*.^52^ However, the origin of H_2_O_2_ in response to photoperiod changes is unclear. In this work, we discovered that CTM communication and mETC play essential roles in the H_2_O_2_ generation in response to photoperiod changes (Fig. 8a), which may result in the disturbance of the chloroplast NADH homeostasis and the triggering of cascade reactions. Our results also demonstrate that the malate shuttle is required for the photoperiod response of plants.

Fatty acids play critical roles in plant development, cell signaling, abiotic stress responses, and pathogen defense.^53–61^ Although it is reasonable to suggest that certain fatty acids or their derivatives in *mod1* might provide the signal inducing ROS and PCD. The results presented here demonstrate that it is the accumulation of reducing equivalent and its transport system that trigger the generation of ROS to a harmful level and induce PCD. These results suggest that the biosynthesis of fatty acids is one of the major reducing equivalent consumers in chloroplasts, and in the cell as a whole.

Although different mutants of the CTM communication system can each reduce the oxidative damage caused by continuous light, they show varying degrees of growth deficiency. The *som430* mutant, which results from a premature stop codon, is embryonic lethal, indicating that the function of *plNAD-MDH* is essential for plant growth. The T-DNA insertion mutant of *DiT1*, *som787-2*, shows remarkably reduced plant size and the point mutation in *mMDH1* leads to a moderate but significant reduced plant size in *som328*. It is notable that the forward genetic screen for *mod1* suppressors did not identify the mitochondrial malate/OAA transporter, which may be due to gene redundancy or lethality. Different levels of growth deficiencies in these mutants indicate that a complicated metabolic regulatory network is likely involved for the CTM communication. This needs to be further investigated to fully understand the crucial function of reducing equivalent homeostasis in plant growth and response to photoperiod.

Plants do not encode caspases and Bcl-2 family members that are required for animal apoptosis, however plant and animal PCD do share several similar morphological and biochemical features, such as cell shrinkage, chromatin condensation, nuclear fragmentation, endonuclease release, and common inhibitors,^2,3,29,62,63^ suggesting that both plant and animal PCD pathways may share a conserved mechanism that triggers and regulates PCD.^63^ This hypothesis is supported by evidence that treatment with malate can induce ROS generation and PCD in HeLa cells. This demonstrates that the malate-induced ROS generation and the PCD pathways are conserved and that mMDH is functionally conserved and essential for PCD pathways in both plants and animals (Fig. 8), even though the malate in *mod1*-triggered PCD originates in chloroplasts that do not exist in animal cells and translocation of malate from the cytosol to mitochondria is carried out by the malate-aspartate shuttle in animal cells and the malate-OAA shuttle in plant cells.

## Materials and methods

### Plant materials and cell culture

Seeds of *Arabidopsis thaliana* ecotype Columbia (Col-0), Landsberg *erecta* (L*er*-0) and *mod1* (in Columbia background) were from our own stocks. The T-DNA insertion mutants were from the Nottingham *Arabidopsis* Stock Centre (NASC) and their genotypes were confirmed by PCR analysis. Primers are listed in Supplementary information, Table S1. HeLa cells were cultured in the Dulbecoo’s modified Eagle Medium (DMEM) with 10% (v/v) Fetal Bovine Serum (FBS).

### Plant growth conditions

*Arabidopsis* seeds were surface sterilized and sown on 0.5 × Murashige and Skoog (MS) medium plates containing 1.0% (w/v) sucrose and 0.65% (w/v) agar. After being vernalized at 4 °C for 3 days, seeds were germinated under long-day conditions (16-hr light/8-hr dark) and seedlings (7 days after germination) were transferred to soil and grown under long-day conditions (16-hr light/8-hr dark) with a light intensity of 80 to 120 μmol m^−2^ s^−1^ at 20–22 °C. For response to different photoperiod conditions, seeds of Col-0, *mod1* and *soms* were sown and germinated in soil directly under long-day conditions (16-hr light/8-hr dark), or continuous illumination conditions (24-hr light) with a light intensity of 85 or 55 μmol m^−2^ s^−1^ at 20–22 °C. In screening for suppressors of *mod1*, M_1_ and M_2_ seeds were sown and germinated directly in soil under continuous illumination conditions (24-hr light) with a light intensity of 80 to 120 μmol m^−2^ s^−1^ at 20–22 °C for the phenotype observation.

### Screening for suppressors

To identify the suppressor of *mod1* (*som)*, ~1.5 g *mod1* seeds were mutagenized with ethyl methanesulfonate (EMS) as previously reported.^64^ M_1_ and M_2_ plants with the restored phenotypes were selected under continuous illumination. The mitochondrial complex I activity of *soms* was assayed as described previously.^26,65^ The *soms* without changes of complex I activities were selected for further studies.

### Map-based cloning and whole-genome resequencing

For the map-based cloning of *SOMs*, *mod1* in the Col-0 background was backcrossed 10 times to L*er* to generate *mod1* plants in the isogenic L*er* background (*mod1-*L*er*). To map the causal mutations in the *soms*, F_2_ populations were generated by crossing the *soms* with *mod1-*L*er*. Individual plants showing restored phenotypes were selected from F_2_ populations for primary mapping. DNA from *mod1* and *soms* was prepared for sequencing using an Illumina HiSeq X Ten with a 150 bp paired-end (PE) strategy. The reads were mapped to the *Arabidopsis* reference genome^66^ using Burrows-Wheeler Aligner (BWA) version 0.7.10-r789 with default parameters.^67^ The SNP calling was based on alignment results using the Genome Analysis Toolkit (GATK) version 3.1.1 and Picard package version 1.119.^68^ The SNPs were then compared between *mod1* and *soms*, and all the nonsynonymous mutations in the mapping region of each *som* are listed in Supplementary information, Table S2.

### Plasmid construction and plant transformation

For the genetic complementation test, fragments containing the full-length genomic DNA of *SOM410* and *SOM328* with their own native promoters were amplified, digested and ligated into the binary vector pCambia1300. The fragment containing the full-length genomic DNA of *SOM787* with its native promoter was cloned into *BamH*I- and *Sal*I- digested pCambia1300 using the Seamless Assembly Cloning Kit (Clone Smarter Technologies). At least two independent complemented lines were obtained and they showed similar phenotypes. For the subcellular localization, the fragment containing the full-length CDS of *SOM328* was digested and ligated into a modified pWM101 vector.^69^ Fragments containing the full-length CDS of *SOM410* and *SOM787* were cloned into *BamH*I- and *Sal*I- digested modified pWM101 using the Seamless Assembly Cloning Kit (Clone Smarter Technologies). For CRISPR/Cas9 mutagenesis, single-guide RNAs (sgRNA) binding in the coding sequence of the target gene were designed using the CRISPR-P tool (http://cbi.hzau.edu.cn/cgi-bin/CRISPR),^70^ and cloned into a binary vectors pHEE2A-TRI^71^ digested with *Bsa*I. Two lines with different frameshift mutation were obtained, and showed similar phenotypes. The final binary vectors were introduced into *Agrobacterium tumefaciens* strain EHA105 and transformed into wild-type and mutant plants through the *Agrobacterium*-mediated floral dip method.^72^ Transformants were selected on 0.5 × MS plates containing 20 mg L^−1^ hygromycin. All primers used are listed in Supplementary information, Table S1.

### RNA expression analysis

For semiquantitative and quantitative RT-PCR, leaves of 2- to 3-week-old plants were collected and immediately flash-frozen in liquid nitrogen. Total RNA was extracted using TRIzol (Invitrogen), and treated with the TURBO DNA-*free*^TM^ kit (Invitrogen) to eliminate contaminated genomic DNA from total RNA. A total of 2.5 μg RNA was used for cDNA synthesis using the SuperScript III First-Strand Synthesis System (Invitrogen). The synthesized cDNA was diluted 10-fold and a 2.0 μL aliquot was used for qRT-PCR in the reaction system of SsoFast^TM^ EvaGreen supermix (Bio-Rad). All primer used are provided in Supplementary information, Table S1.

### Detection of ROS

O_2_^−^ and H_2_O_2_ were detected as previously described.^26,73^ Briefly, 3-week-old plants were immersed and infiltrated under vacuum for 10 min with nitroblue tetrazolium (NBT) staining solution (1 mg mL^−1^ NBT, 10 mM potassium phosphate buffer, 10 mM NaN_3_, pH 7.4) and freshly prepared 3,3′-diaminobenzidine (DAB) staining solution (1 mg mL^−1^ DAB, pH 3.8), respectively. After infiltration, stained plants were bleached in acetic acid:glycerol:ethanol (1:1:3, v/v/v) solution at 100 °C for 10 min, and then stored in 95% (v/v) ethanol until scanning.

### Trypan blue staining

Trypan blue staining was performed as previously described.^26,74^ In brief, leaves were immersed with trypan blue staining solution (30 mL ethanol, 10 g phenol, 10 mL H_2_O, 10 mL glycerol, 10 mL lactic acid and 10 mg trypan blue), boiled for 2–3 min, and then cooled down at room temperature for 1 h. The samples were destained by boiling in chloral hydrate solution (2.5 g mL^−1^) for 20 min with 2–3 changes of the chloral hydrate solution at room temperature. Samples were finally stored in 50% (v/v) glycerol until photographs were taken.

### Preparation of polyclonal antibodies

Fragments containing the full-length CDS of *SOM410* and *SOM328* were amplified and subcloned into pET-61-DEST. Recombinant SOM410 and SOM328 proteins were expressed in *Escherichia coli* BL21 cells and used to raise polyclonal antibodies in mouse. 1:3000 dilution of the anti-SOM410 serum and 1:6000 dilution of the anti-SOM328 serum were used for immunoblotting. All primers used are listed in Supplementary information, Table S1.

### Preparation of leaf proteins

For immunoblotting analysis, extraction of total leaf proteins from 3- to 4-week-old plants was performed as previously described.^26^ For the total MDH activity assay, total leaf proteins from 2- to 3-week-old plants were extracted according to the previously reported method.^75^ Leaves were ground in extraction buffer (50 mM HEPES-KOH, pH 7.5, 1 mM EDTA, 5 mM DTT, 10% (v/v) glycerol and 0.5 mM phenylmethylsulfonyl fluoride). The homogenates were centrifuged at 20,000 *g* at 4 °C for 10 min, the supernatant was transferred into a new tube, quantified by the Bio-Rad protein assay, and used for the determination of the total MDH activity.

### Assay of MDH activity

The total MDH activity was assayed as previously described.^36^ Briefly, freshly extracted total leaf proteins were added into a MDH reaction mixture (90 mM KH_2_PO_4_-KOH, pH 7.4, 0.05% (v/v) Triton X-100, 5 mM MgCl_2_, 750 mM OAA and 2 mM NADH), and the absorbance changes at 340 nm caused by the NADH oxidation to NAD^+^ were used to measure total MDH activity.

### Isolation of mitochondria

Mitochondria were isolated as previously described^76^ with slight changes. Approximately 40 g leaves from 4-week-old plants were homogenized with a Warring blender in 160 mL pre-cooled grinding medium (0.3 M sucrose, 25 mM tetrasodium pyrophosphate, 1% (w/v) polyvinylpyrrolidone-40, 2 mM EDTA, 10 mM KH_2_PO_4_, 1% (w/v) bovine serum albumin (BSA), 20 mM sodium L-ascorbate, 1 mM DTT, 5 mM cysteine, pH 7.5) with three 15 s bursts at 30 s intervals. The homogenate was filtered through four layers of Miracloth and centrifuged at 1500×*g* for 5 min and the resulting supernatant was then centrifuged at 20,000*×g* for 15 min. The organelle pellet was washed by repeating the 1500×*g* and 20,000×*g* centrifugation steps in the washing medium (0.3 M sucrose, 10 mM TES, pH 7.5). The resulting pellet of crude organelles was carefully resuspended in 1–2 mL washing medium and gently layered over a Percoll discontinuous gradient consisting of 1.4-mL 18% (v/v) Percoll, 7-mL 27% (v/v) Percoll and 1.4-mL 50% (v/v) Percoll. The gradient was then centrifuged at 40,000×*g* for 45 min. The mitochondrial band that was seen as a yellow-brown band between 27% (v/v) and 50% (v/v) Percoll was carefully collected by pipette. The mitochondria collected were diluted with washing medium and centrifuged at 20,000×*g* for 10 min. After repeating this washing step 3 times, the final pellet was retained in a small volume of washing medium for later experiments.

### Isolation of chloroplasts

Chloroplasts were isolated using a Chloroplast Isolation Kit (Sigma) following the manufacturer’s instructions with slight modification. Briefly, ~30 g leaves from 4-week-old plants were homogenized in the 120-mL pre-cooled isolation medium containing 0.4 M sorbitol, 50 mM HEPES-KOH, pH 7.6, 2 mM EDTA, 0.1% (w/v) sodium L-ascorbate and 0.1% (w/v) BSA, and the homogenate was filtered through four layers of Miracloth and centrifuged at 200*×g* for 3 min at 4 °C to remove unwanted whole cells and cell wall debris. The supernatant was centrifuged at 1000×*g* for 7 min to pellet the chloroplasts, the pellet was carefully resuspended in 1 to 2 mL of the washing medium (0.4 M sorbitol, 50 mM HEPES-KOH, pH 7.6, and 2 mM EDTA), and gently layered on top of a Percoll gradient consisting of 7-mL 40% (v/v) Percoll and 3.5-mL 80% (v/v) Percoll. After centrifugation at 3000×*g* for 15 min, the intact chloroplasts form a band at the interface between the 40% (v/v) and 80% (v/v) Percoll layers. The band was carefully collected, diluted with the washing medium, and centrifuged at 2000×*g* for 3 min. The chloroplasts were further washed twice and finally resuspended in a 0.5-mL washing medium for later experiments.

### Malate measurement

Leaves from 4-week-old plants were ground into fine powder under liquid nitrogen, and ~30 mg of powder was extracted with 600 μL of acetonitrile:chloroform (7:3, v-v) using 20 ng of D_3_-Malice acid as internal standards for quantitation. After ultrasonic-assisted extraction in an ice-water bath for 1 h and centrifugation at 7000×*g* for 5 min, the supernatant was collected. 300 μL H_2_O was then added to the supernatant for two steps of liquid-liquid partitioning. The upper aqueous fractions were pooled and dried under a nitrogen stream. The samples were redissolved in 300 μL H_2_O and filtered through a 0.22 μm membrane for detection. LC-MS/MS detection was performed on a UPLC (Waters) combined with a 5500 Qtrap MS equipped with an ESI source (AB SCIEX), and 5 μL of each sample was injected onto a HSS T3 C18 column.

Measurements of malate concentration in freshly isolated mitochondria and chloroplasts were carried out in the same way. However, when preparing samples all the solvent volumes used were reduced to 1/6.

### Assays of ROS and cell death in HeLa cells

HeLa cells were cultured in Dulbecoo’s modified Eagle Medium (DMEM) with 10% (v/v) Fetal Bovine Serum (FBS), followed by treatment with 50 mM malate (adjust pH to 7.4 by NaOH) for 24 h. For a negative control, HCl was added to DMEM until the pH was the same as the pH of 50 mM malate and the pH adjusted to 7.4 using NaOH. DHE staining was performed as previously described with slight modification.^77^ In brief, cells were washed twice with the Krebs Ringer buffer (KRB, 135 mM NaCl, 5 mM KCl, 1 mM CaCl_2_, 0.4 mM KH_2_PO_4_, 1 mM MgSO_4_, 20 mM HEPES, 5.5 mM Glucose, pH 7.4), followed by incubation for 15 min at 37 °C in KRB containing 2 μM DHE (Invitrogen). Fluorescence images were obtained using a confocal microscope at excitation and emission wavelengths of 552 nm and 605 nm, respectively. TUNEL staining was performed using an in situ cell death detection kit following the manufacture’s instruction (Sigma Aldrich), and cells were counterstained by DAPI. Fluorescence images were obtained using a confocal microscope at excitation wavelengths of 488 nm and 405 nm for TUNEL and DAPI, respectively.

### HeLa cell transfection

MDH2 RNAi knockdown was performed by transfection of MDH2-siRNAs using Lipofectamine 2000 (Invitrogen), and RNAi knockdown for the negative control was performed by transfection of Control-siRNAs using Lipofectamine 2000 (Invitrogen), following the manufacturer’s instructions. Primers used are listed in Supplementary information, Table S1.

## Electronic supplementary material

Supplementary information, Figure S1

Supplementary information, Figure S2

Supplementary information, Figure S3

Supplementary information, Figure S4

Supplementary information, Figure S5

Supplementary information, Figure S6

Supplementary information, Figure S7

Supplementary information, Figure S8

Supplementary information, Figure S9

Supplementary information, Figure S10

Supplementary information, Figure S11

Supplementary information, Figure S12

Supplementary information, Table S1

Supplementary information, Table S2

## Electronic supplementary material

**Supplementary information** accompanies for this paper at 10.1038/s41422-018-0024-8.
