# Supplementary material for: Malate transported from chloroplast to mitochondrion triggers production of ROS and PCD in Arabidopsis thaliana
Source: Cell Res. 2018 Mar 14;28(4):448–61. doi: 10.1038/s41422-018-0024-8 (PMC5939044; doi:10.1038/s41422-018-0024-8)
Supplement: Supplementary file 1 — Supplementary information, Figure S1 [file 41422_2018_24_MOESM1_ESM.pdf]

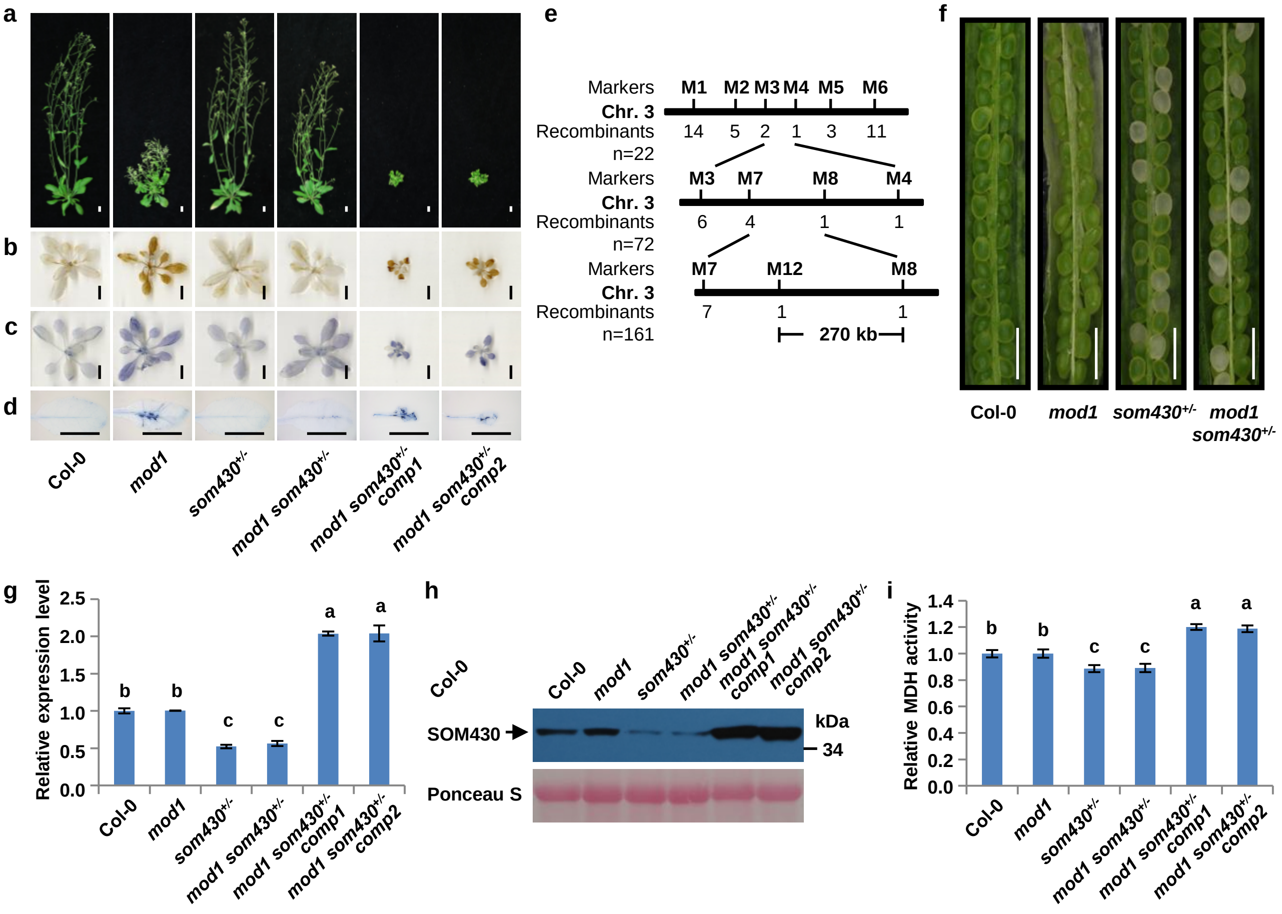

Supplementary information, Figure S1 Cloning and characterization of *SOM430.*

(a) Phenotypes of Col-0, *mod1*, *som430*$^{+/-}$, *mod1 som430*$^{+/-}$, *mod1 som430*$^{+/-}$ *comp1* and *2* (*mod1 som430*$^{+/-}$ complemented line 1 and 2) at 35 days after germination (DAG). Scale bars, 1 cm.

(b) DAB-stained seedlings. Scale bars, 1 cm.

(c) NBT-stained seedlings. Scale bars, 1 cm.

(d) Trypan blue-stained leaves. Scale bars, 1 cm.

(e) Map-based cloning of *SOM430*.

(f) *som430* homozygotes are embryonic lethal. Scale bars, 1 cm.

(g) Transcript levels of *SOM430* in indicated plants, revealed by qRT-PCR using *Actin* as reference. Values are means ± SD (n = 3), and different letters at top of each column indicate a significant difference at $P < 0.05$ determined by Tukey's HSD test.

(h) Protein levels of SOM430 in indicated plants, detected by immunoblotting with anti-SOM410 polyclonal antibodies.

(i) Comparison of total cellular MDH activities in indicated plants. Values are means ± SD (n = 6), and different letters at top of each column indicate a significant difference at $P < 0.05$ determined by Tukey's HSD test.
