# Supplementary material for: Malate transported from chloroplast to mitochondrion triggers production of ROS and PCD in Arabidopsis thaliana
Source: Cell Res. 2018 Mar 14;28(4):448–61. doi: 10.1038/s41422-018-0024-8 (PMC5939044; doi:10.1038/s41422-018-0024-8)
Supplement: Supplementary file 2 — Supplementary information, Figure S2 [file 41422_2018_24_MOESM2_ESM.pdf]

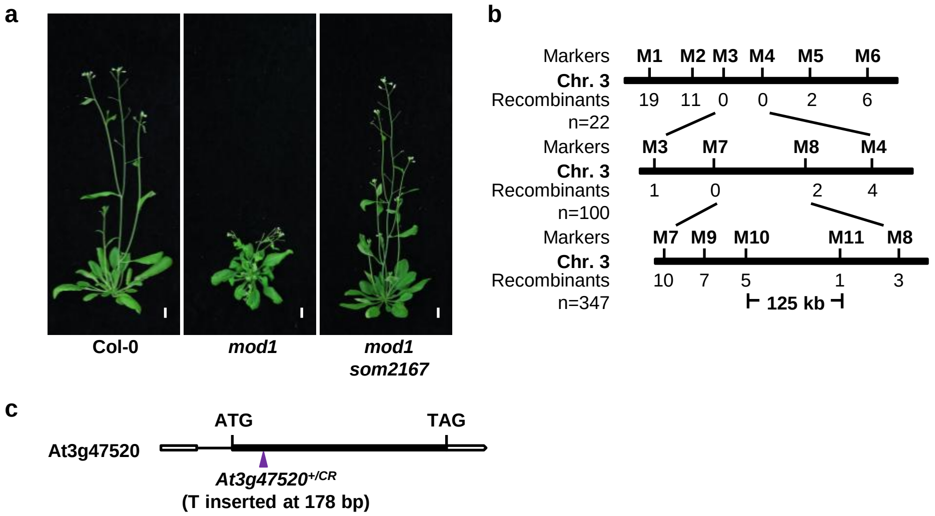

Supplementary information, Figure S2 Map-based cloning of *SOM2167* and the mutation site in *At3g47520*$^{+/CR}$.

(a) Phenotypes of Col-0, *mod1* and *mod1 som2167* at 30 DAG. Scale bars, 1 cm.

(b) Map-based cloning of *SOM2167.*

(c) The mutation site in *At3g47520*$^{+/CR}$ generated by CRISPR/Cas9 mutagenesis.
