# Supplementary material for: Malate transported from chloroplast to mitochondrion triggers production of ROS and PCD in Arabidopsis thaliana
Source: Cell Res. 2018 Mar 14;28(4):448–61. doi: 10.1038/s41422-018-0024-8 (PMC5939044; doi:10.1038/s41422-018-0024-8)
Supplement: Supplementary file 4 — Supplementary information, Figure S4 [file 41422_2018_24_MOESM4_ESM.pdf]

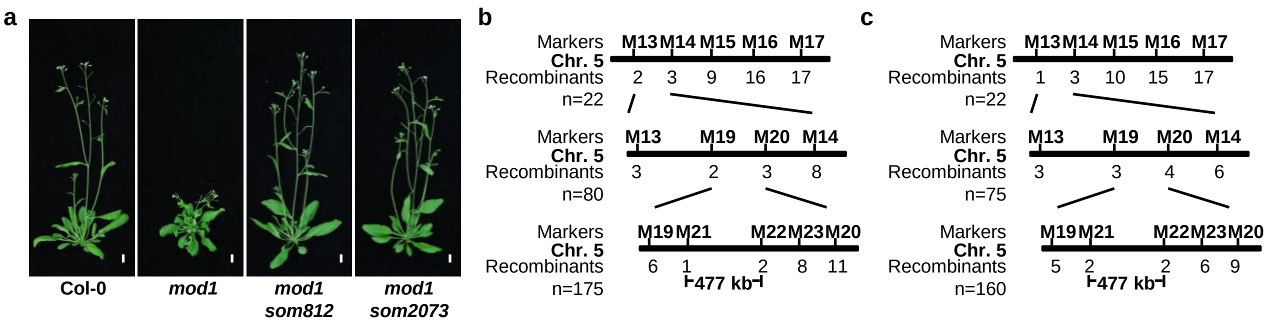

Supplementary information, Figure S4 Map-based cloning of *SOM812* and *SOM2073.*

(a) Phenotypes of Col-0, *mod1*, *mod1 som812* and *mod1 som2073* at 30 DAG. Scale bars, 1 cm.

(b) Map-based cloning of *SOM812.*

(c) Map-based cloning of *SOM2073.*
