# Supplementary material for: Malate transported from chloroplast to mitochondrion triggers production of ROS and PCD in Arabidopsis thaliana
Source: Cell Res. 2018 Mar 14;28(4):448–61. doi: 10.1038/s41422-018-0024-8 (PMC5939044; doi:10.1038/s41422-018-0024-8)
Supplement: Supplementary file 5 — Supplementary information, Figure S5 [file 41422_2018_24_MOESM5_ESM.pdf]

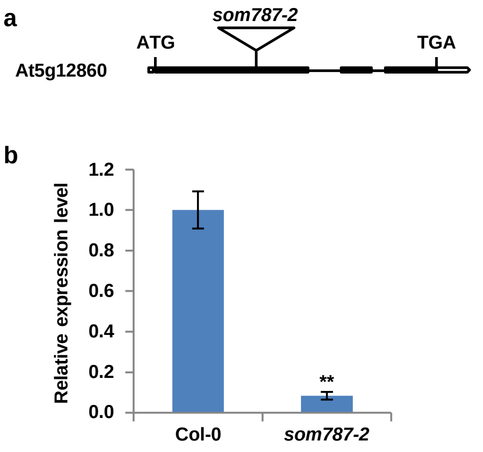

Supplementary information, Figure S5 Characterization of *som787-2*.
(a) The T-DNA insertion site of *som787-2*.
(b) Transcript levels of *SOM787* in Col-0 and *som787-2*, revealed by qRT-PCR using *Actin* as reference. Values are means ± SD (n = 3). The asterisks represent significant difference determined by Student's t test. **P < 0.01.
