# Supplementary material for: Malate transported from chloroplast to mitochondrion triggers production of ROS and PCD in Arabidopsis thaliana
Source: Cell Res. 2018 Mar 14;28(4):448–61. doi: 10.1038/s41422-018-0024-8 (PMC5939044; doi:10.1038/s41422-018-0024-8)
Supplement: Supplementary file 6 — Supplementary information, Figure S6 [file 41422_2018_24_MOESM6_ESM.pdf]

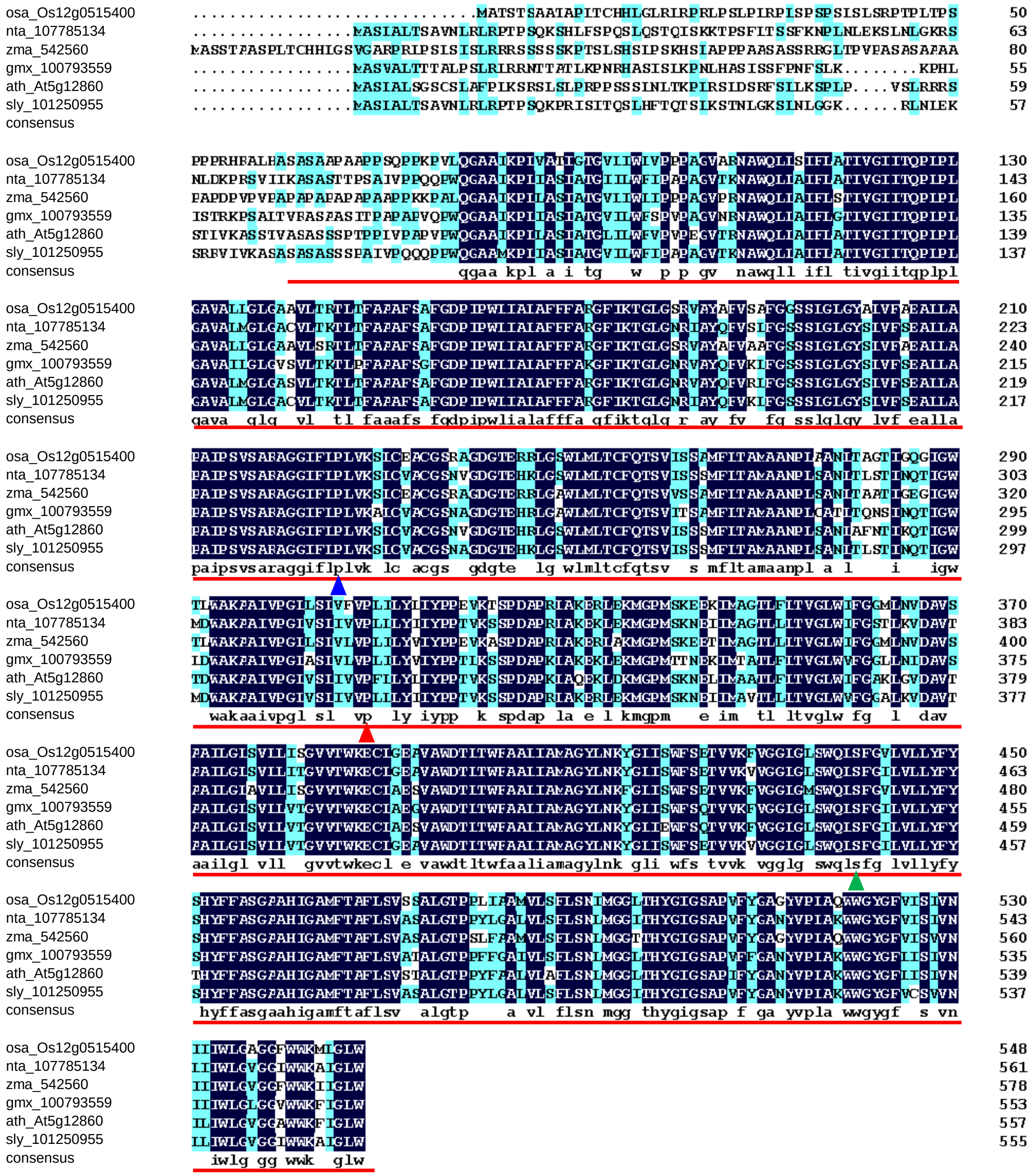

Supplementary information, Figure S6 Amino acid sequence alignments of SOM787.

Amino acid sequence alignments of SOM787 in *Arabidopsis thaliana* (ath), *Zea mays* (zma), *Solanum lycopersicum* (sly), *Nicotiana tabacum* (nta), *Glycine max* (gmx), and *Oryza sativa* (osa). The red line indicates the dicarboxylate transporter domain. Dark blue and cyan shading indicate 100% and > 50% conserved amino acid residues, respectively. The red, blue and green triangles indicate the mutation sites of *som787*, *som812* and *som2073*, respectively.
