# Supplementary material for: Malate transported from chloroplast to mitochondrion triggers production of ROS and PCD in Arabidopsis thaliana
Source: Cell Res. 2018 Mar 14;28(4):448–61. doi: 10.1038/s41422-018-0024-8 (PMC5939044; doi:10.1038/s41422-018-0024-8)
Supplement: Supplementary file 7 — Supplementary information, Figure S7 [file 41422_2018_24_MOESM7_ESM.pdf]

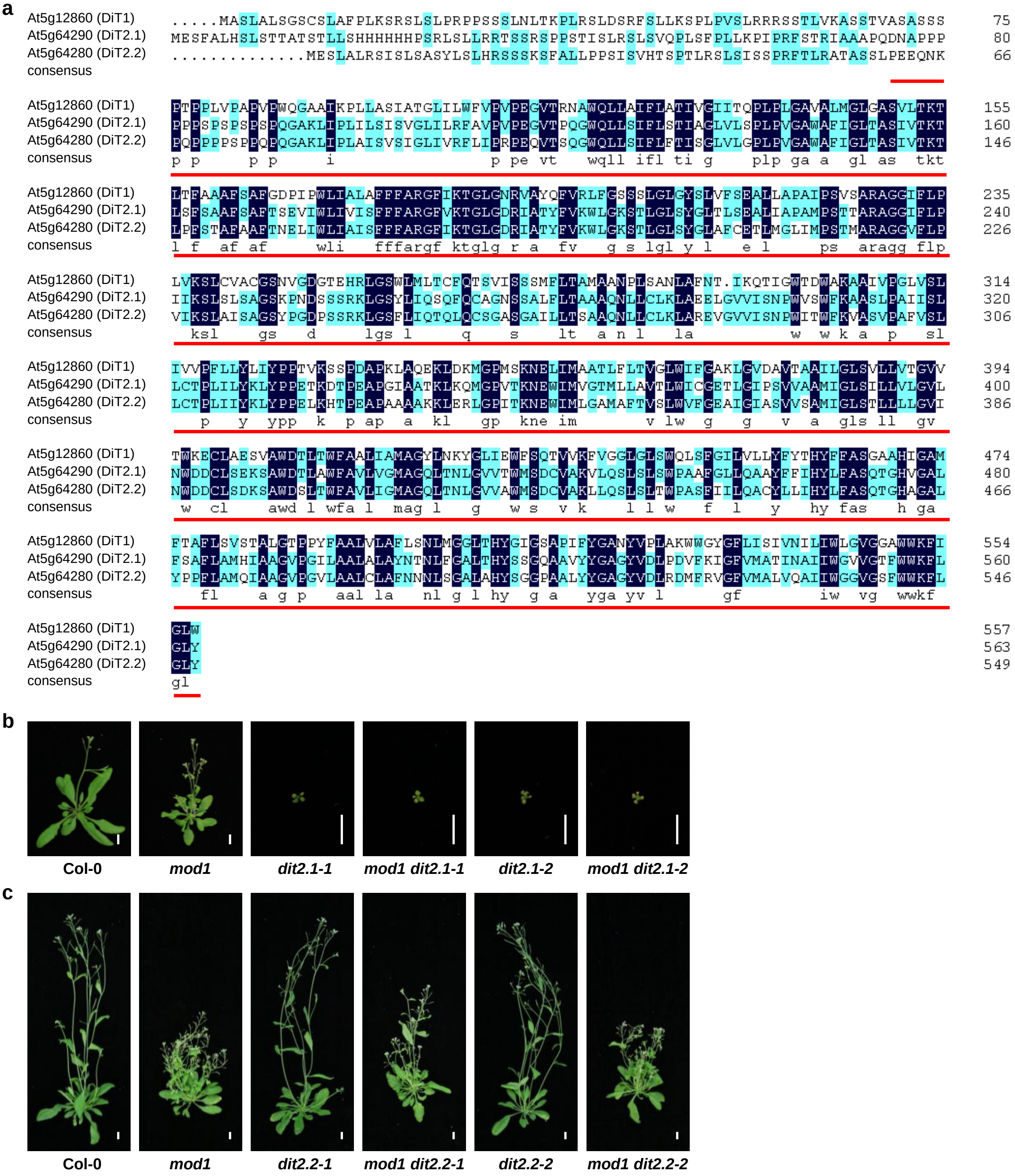

Supplementary information, Figure S7 *dit2.1* and *dit2.2* fail to suppress *mod1* phenotypes.

(a) Amino acid sequence alignments of DiT proteins in *Arabidopsis*. The red line indicates the dicarboxylate transporter domain. Dark blue and cyan shading indicate 100% and 67% conserved amino acid residues, respectively. DiT1, At5g12860; DiT2.1, At5g64290; DiT2.2, At5g64280.

(b) Phenotypes of Col-0, *mod1*, *dit2.1-1*, *mod1 dit2.1-1*. *dit2.1-2*, and *mod1 dit2.1-2* at 26 DAG. N663245; *dit2.1-2*, N669411. Scale bars, 1 cm.

(c) Phenotypes of Col-0, *mod1*, *dit2.2-1*, *mod1 dit2.2-1*. *dit2.2-2*, and *mod1 dit2.2-2* at 35 DAG. *dit2.2-1*, N661169; *dit2.2-2*, N655976. Scale bars, 1 cm.
