# Supplementary material for: Malate transported from chloroplast to mitochondrion triggers production of ROS and PCD in Arabidopsis thaliana
Source: Cell Res. 2018 Mar 14;28(4):448–61. doi: 10.1038/s41422-018-0024-8 (PMC5939044; doi:10.1038/s41422-018-0024-8)
Supplement: Supplementary file 8 — Supplementary information, Figure S8 [file 41422_2018_24_MOESM8_ESM.pdf]

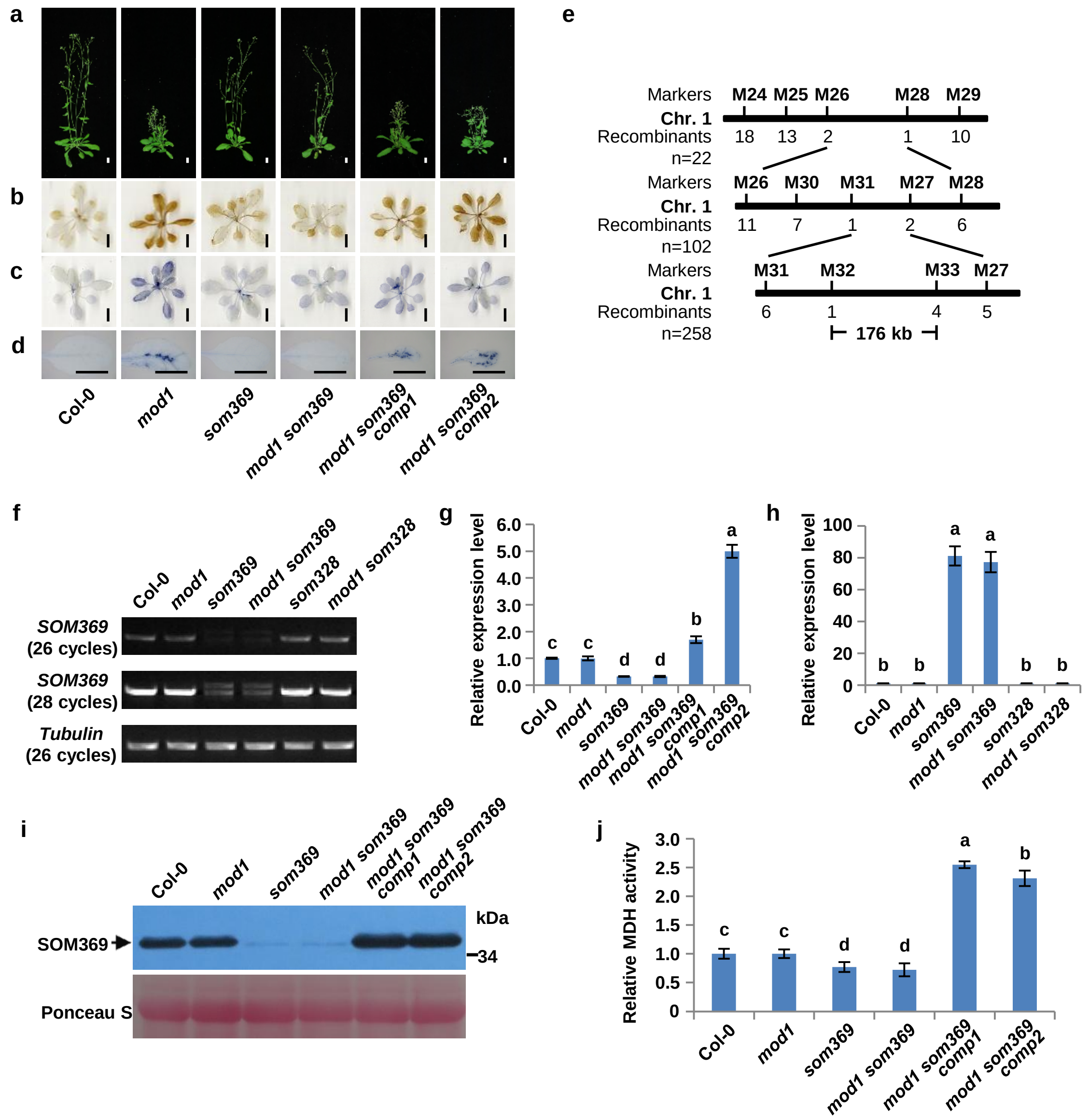

Supplementary information, Figure S8 Cloning and characterization of *SOM369.*

(a) Phenotypes of Col-0, *mod1*, *som369*, *mod1 som369*, *mod1 som369 comp1* and *2* (*mod1 som369* complemented line 1 and 2) at 35 DAG. Scale bars, 1 cm.

(b) DAB-stained seedlings. Scale bars, 1 cm.

(c) NBT-stained seedlings. Scale bars, 1 cm.

(d) Trypan blue-stained leaves. Scale bars, 1 cm.

(e) Map-based cloning of *SOM369*.

(f) Transcript levels of *SOM369* revealed by reverse transcription (RT)-PCR using *Tubulin* as reference.

(g) Transcript levels of *SOM369* revealed by qRT-PCR using *Actin* as reference. Values are means ± SD (n = 3), and different letters at top of each column indicate a significant difference at $P < 0.05$ determined by Tukey's HSD test.

(h) The expression levels of the transcript containing *SOM369 intron 1* revealed by qRT-PCR using *Actin* as reference. Values are means ± SD (n = 3), and different letters at top of each column indicate a significant difference at $P < 0.05$ determined by Tukey's HSD test.

(i) Protein levels of SOM369 in indicated plants detected by immunoblotting with anti-SOM328 polyclonal antibodies.

(j) Comparison of total cellular MDH activities in indicated plants. Values are means ± SD (n = 6), and different letters at top of each column indicate a significant difference at $P < 0.05$ determined by Tukey's HSD test.
