# Supplementary material for: Malate transported from chloroplast to mitochondrion triggers production of ROS and PCD in Arabidopsis thaliana
Source: Cell Res. 2018 Mar 14;28(4):448–61. doi: 10.1038/s41422-018-0024-8 (PMC5939044; doi:10.1038/s41422-018-0024-8)
Supplement: Supplementary file 9 — Supplementary information, Figure S9 [file 41422_2018_24_MOESM9_ESM.pdf]

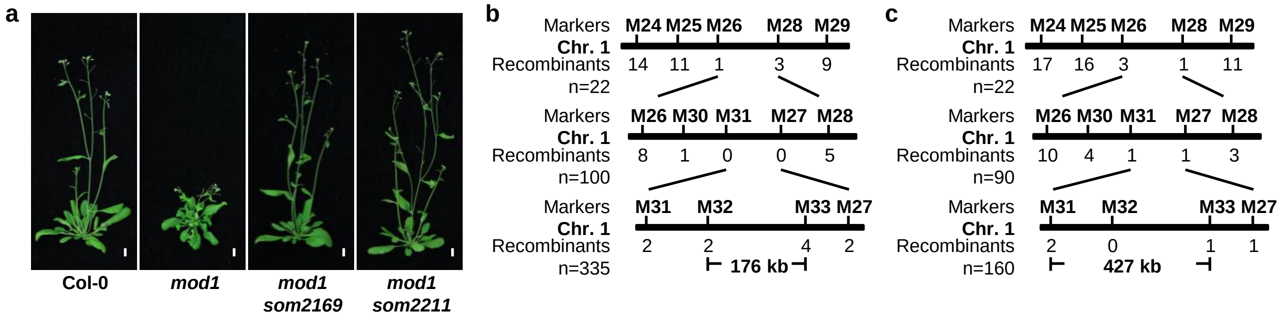

Supplementary information, Figure S9 Map-based cloning of *SOM2169 and SOM2211.*

(a) Phenotypes of Col-0, *mod1*, *mod1 som2169* and *mod1 som2211* at 30 DAG. Scale bars, 1 cm.

(b) Map-based cloning of *SOM2169*.

(c) Map-based cloning of *SOM2211.*
