# Supplementary material for: Malate transported from chloroplast to mitochondrion triggers production of ROS and PCD in Arabidopsis thaliana
Source: Cell Res. 2018 Mar 14;28(4):448–61. doi: 10.1038/s41422-018-0024-8 (PMC5939044; doi:10.1038/s41422-018-0024-8)
Supplement: Supplementary file 10 — Supplementary information, Figure S10 [file 41422_2018_24_MOESM10_ESM.pdf]

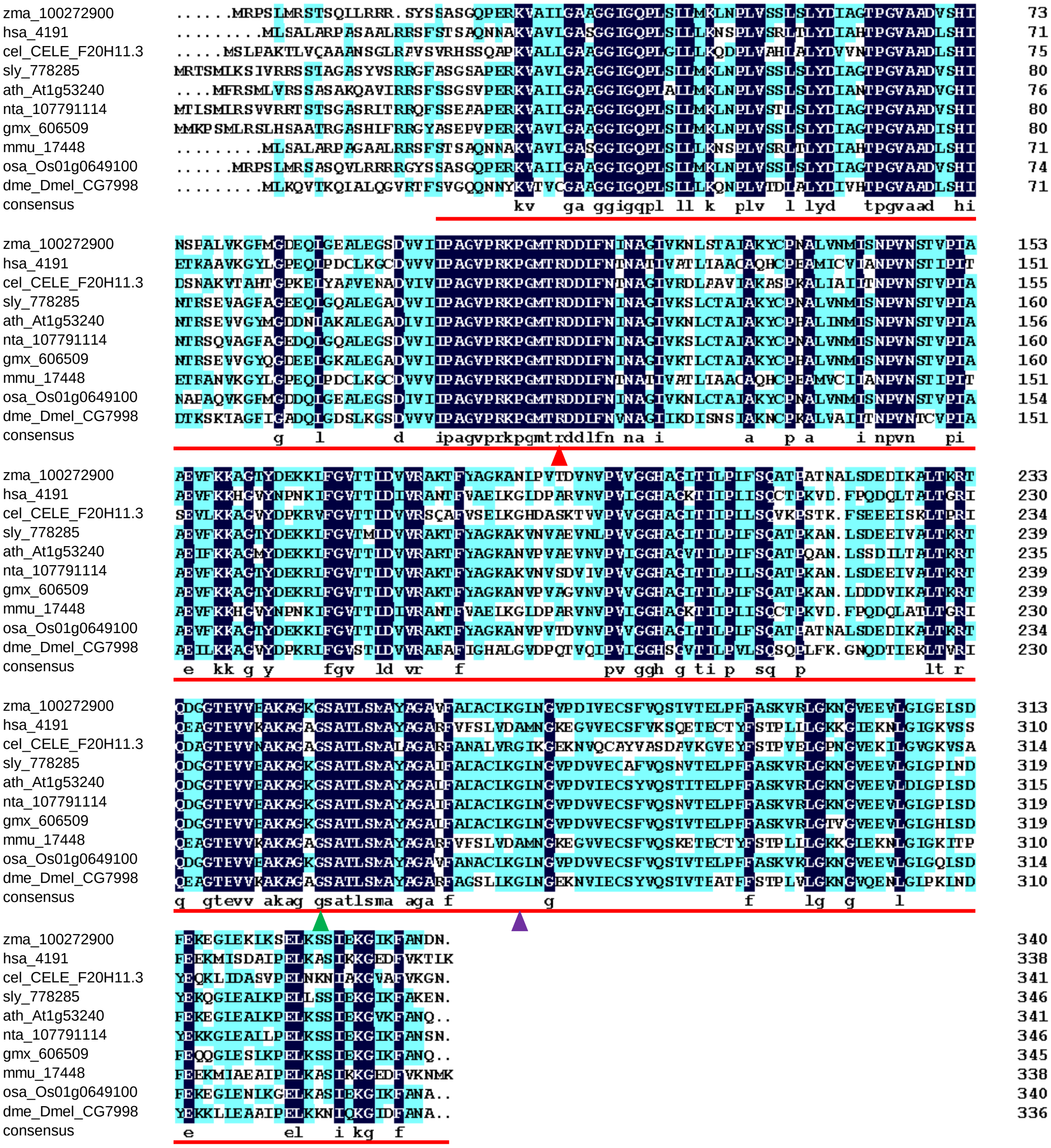

Supplementary information, Figure S10 Amino acid sequence alignments of SOM328.
Amino acid sequence alignments of SOM328 in *Arabidopsis thaliana* (ath), *Zea mays* (zma), *Solanum lycopersicum* (sly), *Nicotiana tabacum* (nta), *Glycine max* (gmx), *Oryza sativa* (osa), *Homo sapiens* (hsa), *Caenorhabditis elegans* (cel), *Mus musculus* (mmu), and *Drosophila melanogaster* (dme). The red line indicates the malate dehydrogenase domain. Dark blue and cyan shading indicate 100% and > 50% conserved amino acid residues, respectively. The red, green and purple triangles indicate the mutation sites of *som328*, *som2169* and *som2211*, respectively.
