# Supplementary material for: Malate transported from chloroplast to mitochondrion triggers production of ROS and PCD in Arabidopsis thaliana
Source: Cell Res. 2018 Mar 14;28(4):448–61. doi: 10.1038/s41422-018-0024-8 (PMC5939044; doi:10.1038/s41422-018-0024-8)
Supplement: Supplementary file 11 — Supplementary information, Figure S11 [file 41422_2018_24_MOESM11_ESM.pdf]

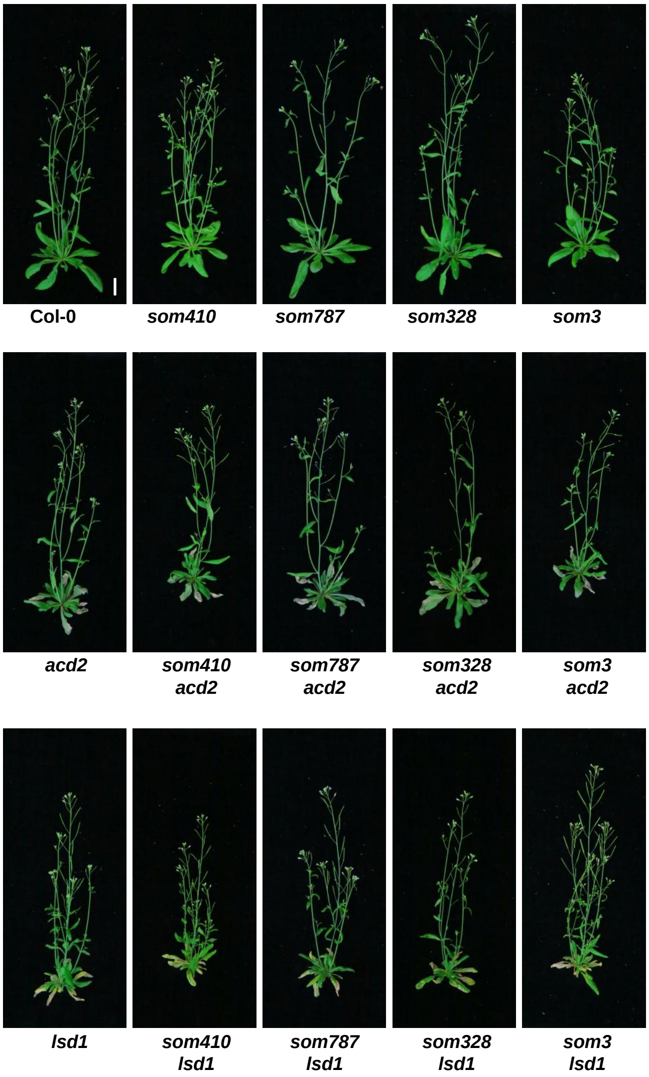

Supplementary information, Figure S11 *som410*, *som787*, *som328* and *som3* fail to suppress the cell death in *acd2* and *lsd1.*

Phenotypes of Col-0, *soms*, *acd2*, *lsd1*, and the double mutants of *som acd2* and *som lsd1* at 32 DAG. Scale bars, 1 cm.
