# Supplementary material for: Malate transported from chloroplast to mitochondrion triggers production of ROS and PCD in Arabidopsis thaliana
Source: Cell Res. 2018 Mar 14;28(4):448–61. doi: 10.1038/s41422-018-0024-8 (PMC5939044; doi:10.1038/s41422-018-0024-8)
Supplement: Supplementary file 12 — Supplementary information, Figure S12 [file 41422_2018_24_MOESM12_ESM.pdf]

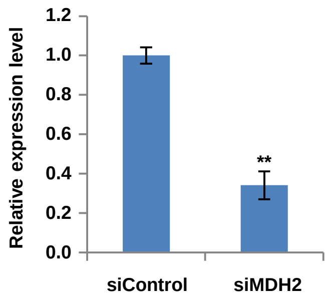

Supplementary information, Figure S12 The expression level of *MDH2* in siMDH2. The expression levels of *MDH2* in siMDH2 revealed by qRT-PCR. Values are means ± SD (n = 3). The asterisks represent significant difference determined by Student's t test. **$P < 0.01$.
