# Supplementary material for: Malate transported from chloroplast to mitochondrion triggers production of ROS and PCD in Arabidopsis thaliana
Source: Cell Res. 2018 Mar 14;28(4):448–61. doi: 10.1038/s41422-018-0024-8 (PMC5939044; doi:10.1038/s41422-018-0024-8)
Supplement: Supplementary file 13 — Supplementary information, Table S1 [file 41422_2018_24_MOESM13_ESM.pdf]

Supplementary information, Table S1 Oligos Used in This Study

Primer	Sequence (5'-3')
**Primers for qRT-PCR**	
Actin-F	GCACCACCTGAAAGGAAGTACA
Actin-R	CGATTCCTGGACCTGCCTCATC
SOM410-F	TGGTACTGAGGTTGTTGAT
SOM410-R	GAGCACGAAGAGATGACT
SOM787-F	CCTTCTTGTGACAGGTGTTG
SOM787-R	CAGCCATCGCAATGAGAG
SOM328-F	GGAGGAGGTTCTTGACTT
SOM328-R	TCTATGGAGGACTTGAGTTC
SOM369-intron1-F	GCTTATTAGACTTGTGACTTGT
SPOM369-intron1-R	GCTGGAATGATAACGAGATC
**Primers for RT-PCR**	
Tubulin-F	TTTGGAGCCTGGGACTATGGAT
Tubulin-R	ACGGGGGAATGGGATGAGAT
SOM328-F	ATTGGTCAGCCTCTTGCT
SOM328-R	ACTGGGACATTTGCCTTT
**Primers for CRISPR/Cas9 genome-editing**	
SOM410-sgRNA-F	ATTGCCTTAGCCAAGACTCTACG
SOM410-sgRNA-R	AAACCGTAGAGTCTTGGCTAAGG
**Primers for complementation test**	
*SOM410-Eco*RI-F	AAAGAATTCTTACTGCCAACTAATAGACATTAG
*SOM410-Xba*I-R	AAATCTAGAGATTTTTTGGTGATGATTTATA
*SOM328-Eco*RI-F	AAAGAATTCCCTTCTCGTCGGTCTTTC
*SOM328-Xba*I-R	AAATCTAGATAAAAATCTCTTTTACTTTTGGCTG
*SOM787-Bam*HI-F	ATTACGAATTCGAGCTCGGTACCCGGGTGTCACTTTACACACCTTTTC
*SOM787-Sal*I-R	GCCAGTGCCAAGCTTGCATGCCTGCAGGTATCAAGTAAAGTGGTGGTCTC
**Primers for subcellular location**	
*SOM410-Bam*HI-F	AGCTTTCGCGAGCTCGGTACCCGGGGATCCATGGCAACAGCAACATCAGCTTC
*SOM410-Sal*I-R	CTCGCCCTTGCTCACCATAAGCTTGTCGACGTTAGCTGCTGCAGCAGCTGGTTTG
*SOM328-Bam*HI-F	AAAGGATCCATGTTCAGATCTATGCTC
*SOM328-Sal*I-R	AAAGTCGACCTGGTTGGCAAACTTGAC
*SOM787-Bam*HI-F	AGCTTTCGCGAGCTCGGTACCCGGGGATCCATGGCGTCTCTCGCTCTCTCCGGCTCCT[illegible]
*SOM787-Sal*I-R	CTCGCCCTTGCTCACCATAAGCTTGTCGACCCACAAGCCAATGAACTTCCACCAGGCAC
**Primers for polyclonal antibodies**	
SOM410-F	GGGGACAAGTTTGTACAAAAAAGCAGGCTTAATGGCAACAGCAACATCAGCTTC
SOM410-R	GGGGACCACTTTGTACAAGAAAGCTGGGTAGTTAGCTGCTGCAGCAGCTGGT
SOM328-F	GGGGACAAGTTTGTACAAAAAAGCAGGCTTAATGTTCAGATCTATGCTCGTCC
SOM328-R	GGGGACCACTTTGTACAAGAAAGCTGGGTACTGGTTGGCAAACTTGACTCCC
**Primers for T-DNA insertion mutants genotyping**	
som787-2-F	GATTTAATTCCAAGCGAAGCC

som787-2-R	ACTCCCACAAGCAACACAGAG
som328-2-F	TAGTAACCCCAGCATGACCAC
som328-2-R	CTATGCTCGTCCGATCTTCTG
DiT2.1-1-F	CTTAATCTGAGGGCTGCTTTG
DiT2.1-1-R	TCAGGCAGATCAACATAACCC
DiT2.1-2-F	TTTGATTCATGATTTGGCTCC
DiT2.1-2-R	TCAGGCAGATCAACATAACCC
DiT2.2-1-F	TACATACCCAGCTGCAGATCC
DiT2.2-1-R	ACCCATCTTCCAGAAAATTGG
DiT2.2-2-F	GTTGTTGAAAGCCAAGCAAAG
DiT2.2-2-R	TTGCCGGTGATTAAATCTTTG
LSD1-F	CTGGGATTTGTAAAGCAGCTG
LSD1-R	TCAAGTTCCATGGAGCAAAAG
**Primers for RNAi knockdown**	
MDH2-siRNA-F	CCCUUGUGGAUGCAAUGAATT
MDH2-siRNA-R	UUCAUUGCAUCCACAAGGGTT
Control-siRNA-F	UUCUCCGAACGUGUCACGUTT
Control-siRNA-R	ACGUGACACGUUCGGAGAATT
**Primers for map-based cloning**	
M1-F	CTCTGTCACTCTTTTCCTCTGG
M1-R	CATGCAATTTGCATCTGAGG
M2-F	CCCCGAGTTGAGGTATT
M2-R	GAAGAAATTCCTAAAGCATTC
M3-F	AGTGCAAAAGAGCTGTATAA
M3-R	ATTTGTGGGATTCTTTCTCA
M4-F	GCACACATTTGTTATGTTAT
M4-R	TAGCCGAACCGCACCTTT
M5-F	GTTCATTAAACTTGCGTGTGT
M5-R	TACGGTCAGATTGAGTGATTC
M6-F	ATGGAGAAGCTTACACTGATC
M6-R	TGGATTTCTTCCTCTCTTCAC
M7-F	AGAAGCTTTGTGTCCAGTAG
M7-R	GAACCTGGTGTAACGTGTTG
M8-F	AAACAAATTCCAATTTACAA
M8-R	CGCCACAAATTTAAGCCT
M9-F	TCCACAACCAAAGAAAGA
M9-R	TTGATGAGTCTGAATGGC
M10-F	CTTAGTCCTCGAATCCAT
M10R	GAAGCCATTCTAATAACC
M11-F	TAGTCTTGTTGATGGCTTGA
M11-R	TCCCTCGTTAGGTTTCTG
M12-F	AATGGACTTCCCCAAGAAA
M12-R	GTAAATAAGACGGAGAAGAG

M13-F	CCACTTGTTTCTCTCTCTAG
M13-R	TATCAACAGAAACGCACCGAG
M14-F	CCATTGGAGATGGTCTTAC
M14-R	CTTAAGCTCCAAGCTATCA
M15-F	AAACTCGAGAGTTTTGTCTAGATC
M15-R	CTCAGAGAATTCCCAGAAAAATCT
M16-F	CAGACGTATCAAATGACAAATG
M16-R	GACTACTGCTCAAACTATTCGG
M17-F	TTGGATCTGATATGTGATG
M17-R	TCGATAAATGCAAAAATAC
M18-F	AGGCACCAAAGAAACAAG
M18-R	CGTTGTGTTCATTTATGAC
M19-F	GGGGACATTTAGGTGGTATC
M19-R	CATCGCCGCCAATACCTC
M20-F	TAAATTAAGGCCACAACTG
M20-R	CATCGAAAAAGCACTTTAC
M21-F	CAAAAACTTTTGGAGTTTG
M21-R	GTCATTGTTATGCTACTTG
M22-F	CCCAGTCTAACCACGACCAC
M22-R	AATCCCAGTAACCAAACACACA
M23-F	TTCCCAGAGCTTGTTTTG
M23-R	AGTCTAAAAGCGAGAGTATG
M24-F	GGCTTTCTCGAAATCTGTCC
M24-R	TTACTTTTTGCCTCTTGTCATTG
M25-F	CTTTCAAAAGCACATCACA
M25-R	AGGTTTTATTGCTTTTCACA
M26-F	ACATTTTCTCAATCCTTACTC
M26-R	GAGAGCTTCTTTATTTGTGAT
M27-F	TAAAGATACGATTCGTAAGA
M27-R	CTAGCAGGCAAATGAGAC
M28-F	GGCTCCATAAAAAGTGCACC
M28-R	CTGATCTCACGGACAATAGTGC
M29-F	TGTTTTTTAGGACAAATGGCG
M29-R	CTCCAGTTGGAAGCTAAAGGG
M30-F	AATGAAGAATTGGGACTGTT
M30-R	ATATCCTCCTCCTGTGTCTC
M31-F	ACTCCGTCTTCCAGAGTT
M31-R	TAGTTATTTCGGGTGATTAG
M32-F	CACGTTGTAGAGAAGTAATA
M32-R	GCCAGTTAATAGAATTTGTT
M33-F	CTTTCTTTTAACGACGATAT
M33-R	ATTGTTCAATGTTCACCA
