# Supplementary material for: Malate transported from chloroplast to mitochondrion triggers production of ROS and PCD in Arabidopsis thaliana
Source: Cell Res. 2018 Mar 14;28(4):448–61. doi: 10.1038/s41422-018-0024-8 (PMC5939044; doi:10.1038/s41422-018-0024-8)
Supplement: Supplementary file 14 — Supplementary information, Table S2 [file 41422_2018_24_MOESM14_ESM.pdf]

Supplementary information, Table S2 Nonsynonymous mutations in the mapping region of each *som*

ID	Chromosome	Position	Mutation	Gene ID	Gene name
som410	Chr3	17513925	C>T	AT3G47520	*plNAD-MDH*
som430	Chr3	17504882	G>A	AT3G47500	*CDF3*
som430	Chr3	17514056	C>T	AT3G47520	*plNAD-MDH*
som430	Chr3	17628913	C>T	AT3G47780	*ATATH6*
som2167	Chr3	17493718	C>T	AT3G47470	*LHCA4*
som2167	Chr3	17493742	C>T	AT3G47470	*LHCA4*
som2167	Chr3	17493748	C>T	AT3G47470	*LHCA4*
som2167	Chr3	17493958	G>A	AT3G47470	*LHCA4*
som2167	Chr3	17514570	C>T	AT3G47520	*plNAD-MDH*
som787	Chr5	4060968	G>A	AT5G12860	*DiT1*
som812	Chr5	4061216	C>T	AT5G12860	*DiT1*
som2073	Chr5	4060255	G>A	AT5G12860	*DiT1*
som328	Chr1	19841825	C>T	AT1G53200	
som328	Chr1	19856322	C>T	AT1G53240	*mMDH1*
som369	Chr1	19856559	C>T	AT1G53240	*mMDH1*
som2169	Chr1	19855429	C>T	AT1G53240	*mMDH1*
som2169	Chr1	19868333	C>T	AT1G53282	
som2211	Chr1	19706484	C>T	AT1G52905	
som2211	Chr1	19855275	C>T	AT1G53240	*mMDH1*
